# Meta-Analysis of Public Microarray Datasets Reveals Voltage-Gated Calcium Gene Signatures in Clinical Cancer Patients

**DOI:** 10.1371/journal.pone.0125766

**Published:** 2015-07-06

**Authors:** Chih-Yang Wang, Ming-Derg Lai, Nam Nhut Phan, Zhengda Sun, Yen-Chang Lin

**Affiliations:** 1 Department of Anatomy, University of California San Francisco, San Francisco, California, United States of America; 2 Department of Biochemistry and Molecular Biology, College of Medicine, National Cheng Kung University, Tainan, Taiwan; 3 Institute of Basic Medical Sciences, College of Medicine, National Cheng Kung University, Tainan, Taiwan; 4 Faculty of Applied Sciences, Ton Duc Thang University, Ho Chi Minh, Vietnam; 5 Department of Radiology, University of California San Francisco, San Francisco, California, United States of America; 6 Graduate Institute of Biotechnology, Chinese Culture University, Taipei, Taiwan; Deutsches Krebsforschungszentrum, GERMANY

## Abstract

Voltage-gated calcium channels (VGCCs) are well documented to play roles in cell proliferation, migration, and apoptosis; however, whether VGCCs regulate the onset and progression of cancer is still under investigation. The VGCC family consists of five members, which are L-type, N-type, T-type, R-type and P/Q type. To date, no holistic approach has been used to screen VGCC family genes in different types of cancer. We analyzed the transcript expression of VGCCs in clinical cancer tissue samples by accessing ONCOMINE (www.oncomine.org), a web-based microarray database, to perform a systematic analysis. Every member of the VGCCs was examined across 21 different types of cancer by comparing mRNA expression in cancer to that in normal tissue. A previous study showed that altered expression of mRNA in cancer tissue may play an oncogenic role and promote tumor development; therefore, in the present findings, we focus only on the overexpression of VGCCs in different types of cancer. This bioinformatics analysis revealed that different subtypes of VGCCs (CACNA1C, CACNA1D, CACNA1B, CACNA1G, and CACNA1I) are implicated in the development and progression of diverse types of cancer and show dramatic up-regulation in breast cancer. CACNA1F only showed high expression in testis cancer, whereas CACNA1A, CACNA1C, and CACNA1D were highly expressed in most types of cancer. The current analysis revealed that specific VGCCs likely play essential roles in specific types of cancer. Collectively, we identified several VGCC targets and classified them according to different cancer subtypes for prospective studies on the underlying carcinogenic mechanisms. The present findings suggest that VGCCs are possible targets for prospective investigation in cancer treatment.

## Introduction

In the last few decades, cancer has become a focal cause of death worldwide. Until recently, therapeutic methods applied as cancer treatments (primarily surgery, chemotherapy, radiation therapy) had not changed much from 40 years ago. Although different research approaches have been taken to enhance the survival rate and life quality of cancer patients, much effort and many more trials are still needed to accelerate and facilitate cancer treatment.

Ion channels are well documented as novel potential therapeutic targets in cancer treatment due to their integration with many cancer features such as cell proliferation, apoptosis, metastatic capability and migration [1]. Calcium (Ca^2+^) is the key player in cell proliferation, activating or inhibiting various intracellular enzymes in numerous compartments including the cytosol, organelles, and nucleus. Intracellular Ca^2+^ levels, through calmodulin, regulate many different kinases, phosphatases, cyclases, esterases and ion channels. A number of mechanisms involving plasma membrane ion channels and ion exchangers associated with the endoplasmic reticulum and nuclear envelope calcium stores control the levels of free Ca^2+^ in the protoplasm [2, 3]. The impact of changes in Ca^2+^ can be specifically determined by the location, extent, duration, and timing of intracellular Ca^2+^ oscillations. For instance, slight variations in Ca^2+^ could regulate specific cell functions, whereas a substantial alteration of Ca^2+^ could be responsible for cell proliferation and motility or even cell apoptosis [4].

Calcium channels can be classified into two main types: voltage-gated calcium channels (VGCCs) and ligand-gated calcium channels. The L-type [5, 6], N-Type [7], P-type [8–10], T-type [11–13] and R-type [14, 15] calcium channels that constitute the VGCC family are involved in the development of various types of cancer (Table 1). In addition, ligand-gated calcium channels regulate many processes occurring at the onset of cancer such as activation of the IP3 receptor [16] and ryanodine [17].

**Table 1:** Voltage-gated calcium channel localization and functions.

Channel	Current	Associated subunits	Expression detected	General Cellular functions	References
Cav1.1 (CACNA1S)	L	α2δ, β, γ	Brain, Leukemia	Excitation-contraction coupling	[18–23]
Cav1.2 (CACNA1C)	L	α2δ, β, γ	Colorectal, Gastric, Pancreas, Sarcoma, Leukemia, Brain, Breast, Uterus, Skin, Prostate	Excitation-contraction coupling	[18–23]
Cav1.3 (CACNA1D)	L	α2δ, β, γ	Prostate, Breast, Colorectal, Bladder, Gastric, Lung, Brain, Uterus, Esophagus	Excitation-contraction coupling	[18–23]
Cav1.4 (CACNA1F)	L	α2δ, β, γ	Testis	Excitation-contraction coupling	[18–23]
Cav2.1 (CACNA1A)	P/Q	α2δ, β, possibly γ	Leukemia, Ovarian, Sarcoma, Brain, Uterus, Ovarian, Lung, Cervix,	Neurotransmitter release; dendritic Ca2+ transients; hormone release	[18, 19, 24–29]
Cav2.2 (CACNA1B)	N	α2δ/β1, β3, β4, possibly γ	Prostate, Breast	Neurotransmitter release; dendritic Ca2+ transients; hormone release	[18, 19, 28, 30–33]
Cav2.3 (CACNA1E)	R	α2δ, β, possibly γ	Esophagus, Uterus	Repetitive firing; dendritic calcium transients	[18, 19, 34–37]
Cav3.1 (CACNA1G)	T	None	Sarcoma, Colorectal, Uterus, Lung, Prostate, Breast	Pacemaking; repetitive firing	[18, 19, 38–43]
Cav3.2 (CACNA1H)	T	None	Renal, Sarcoma, Gastric	Pacemaking; repetitive firing	[18, 19, 38–43]
Cav3.3 (CACNA1I)	T	None	Breast, Sarcoma, Esophagus	Pacemaking; repetitive firing	[18, 19, 38–43]

Microarray technology has introduced an experimental approach without bias into sample screening and data collection, leading to the creation of hypotheses [44]. Although the data from these analyses need to be confirmed by further detailed studies, it nonetheless helps to somehow foresee the trend of information. Genes are usually considered to represent potential cancer markers when they show differential overexpression in a particular cancer. The existing literature contains thousands of mRNA expression profile studies of various cancers, and a large number of datasets have been made publicly available. The proper and full utilization of this huge resource would therefore accelerate the identification of important cancer markers as well as facilitate the development of improved molecular signatures. A previous study showed that altered gene expression in cancer tissue may play an oncogenic role and promote tumor development; therefore, in the present findings, we focus only on the overexpression of VGCCs in different types of cancer. We hypothesized, based on our bioinformatics screening, that an increase in mRNA expression of VGCCs reflects some degree of participation in cancer progression and development. We have explored potential markers of VGCC overexpression in cancer using the web-based ONCOMINE microarray database [45, 46]. The current investigation focused on the novel regulation of calcium channel family members in different types of cancer, with the supposition that these clinical data would provide important hints that will enable further investigation of the roles of these voltage-gated calcium channels in the progression and development of cancer.

## Materials and Methods

The expression of VGCC mRNA in clinical cancer tissues was analyzed by performing a meta-analysis of public microarray data according to PRISMA guidelines [47, 48] (S1 Table and S1 Fig). We used the web-based microarray database called ONCOMINE (www.oncomine.org) to obtain a systematic analysis of all public cancer microarray data. The website document “ONCOMINE Platform Overview Q1 2014” indicates that this database contains more than 700 independent datasets comprising nearly 90,000 microarray experiments. Most microarray expression analyses define the up and down-expression of genes in nearly every major cancer type as well as in a number of clinical and pathology-based cancer subtypes.

We set threshold criteria to screen potential oncogenes with respect to datasets regulating VGCC transcript expression in cancer tissues [49, 50]. The statistical levels for the screening criteria used in this study were as follows: the fold change must above 1.5, the P-value must be less than 0.05, and the percentile ranking of the gene must be less than 10%. P-values and statistical significance in different types of cancer for differential expression of VGCCs were calculated using the ONCOMINE default algorithms, which included two-tailed Student's t-test and multiple testing corrections. In the present report, a P-value <0.05 indicated a statistically significant difference between samples. We used a fold-change-based benchmark to identify linear model correlation between mRNA levels and VGCC gene expression in cancer tissues relative to normal expression levels in the same tissue section. Only samples with a fold change >1.5 were chosen for inclusion in the investigative procedure. The degree of expression was determined from the gene rank percentile, which typically classified the genes of interest according to p-values. The top 10% of the altered VGCC genes were used in the analytical process. Ultimately, we retained 50 studies integrating 8174 samples (S2 Table and S1 Fig).

To present the collected datasets, samples must be reviewed and grouped into logical sample sets. The analysis types are matched cancer/normal tissue and the numerous molecular subtypes, biomarker status, treatment responses, and other miscellaneous comparisons. After the classification of logical analyses, each gene was assessed using different statistical analyses such as Student’s t-test and Pearson’s correlation depending on how many classes of ordinal analyses were found. These tests were completed using the R statistical computing package (http://www.r-project.org). Tests were carried out as one-sided or two-sided based on the type of expression analysis. To rationalize the numerous hypothesis assessments, we computed Q values using the following equation: Q = NP / R where P is the P-value, N is the number of genes analyzed, and R is the sorted rank of the P-value [45, 46]. The expression of the gene CACNA1A in ovarian [51], breast [52], lung [53] and gastric cancer was analyzed using the Kaplan-Meier Plotter (http://kmplot.com/analysis/) database, which consists of a pool of gene expression and clinical data. Up to the present, this database covers information on 22,277 genes and their influence on survival in 4,142, 1,648, 765 and 2,437 patients with breast, ovarian, gastric and lung cancer, respectively. We focused our analysis on overall survival patient information. There are two groups of patient samples, which are higher and lower expression levels. A Kaplan-Meier survival plot was employed to compare the expression of CACNA1A in those two groups. The hazard ratio with 95% confidence intervals and log rank *p value* was also computed (S2 and S3 Figs).

## Results and Discussion

### 1. Voltage-gated calcium channel family promotes cancer development

The dynamic balance between extracellular and intracellular Ca2+ generally regulates calcium signals [54]. This oscillation plays a crucial role in a cell’s ability to recommence the cell cycle, to stimulate DNA synthesis at the G1/S transition, and to enter into mitosis during M phase of the cell cycle [4]. The potential of the so-called T-type calcium channel subtype to moderate the intracellular Ca2+ level has made this channel a focus for regulation in malignant tumor cells [4].

Calcium channels are key players in the cell proliferation process. T-type calcium channels have recently drawn attention as potential therapeutic targets in cancer treatment. A T-type calcium channel inhibitor leads to cell growth inhibition and apoptosis in HCT116 cells [55]. It is also well documented that T-type selective properties have anti-proliferative effects in malignant tumor cells [56]. T-type channels are well documented to be involved in cell growth and differentiation, to be over-expressed in various stages of tumors, and to participate in calcium-mediated cell growth [55–58]. In addition, T-type calcium channels are broadly expressed in different types of cancer and play a key role in cell proliferation [57, 59]. Several calcium channel blockers, such as verapamil [60], nifedipine [61], TH-1177 [62], 2-APB [63], and SK&F 96365 [64], have been confirmed to inhibit receptor-gated calcium channels, but the particular subtypes of calcium channel have not been investigated. Instead, the involvement of calcium channels in cell growth has been highlighted. We hypothesize that focusing on specific calcium channel subtypes may identify the ones that are controlling the proliferation of different cell types.

Cell migration plays a vital role in various physiological processes such as neural crest cell immigration, leukocyte discharge from the vasculature, and the relocation of fibroblasts during wound healing. Cell migration is also extremely pivotal in metastatic diseases and the development of malignancies. The fundamental mechanism that promotes cell migration is indistinguishable with respect to different cell types. Calcium channel types correlate with various types of cancer, e.g., breast [65], prostate [66], and ovarian [67] cancer. Ca2+ channel activity also triggers oxidative phosphorylation, programmed cell death, and alterations in the apoptosis signaling pathway [68].

**Fig 1:**
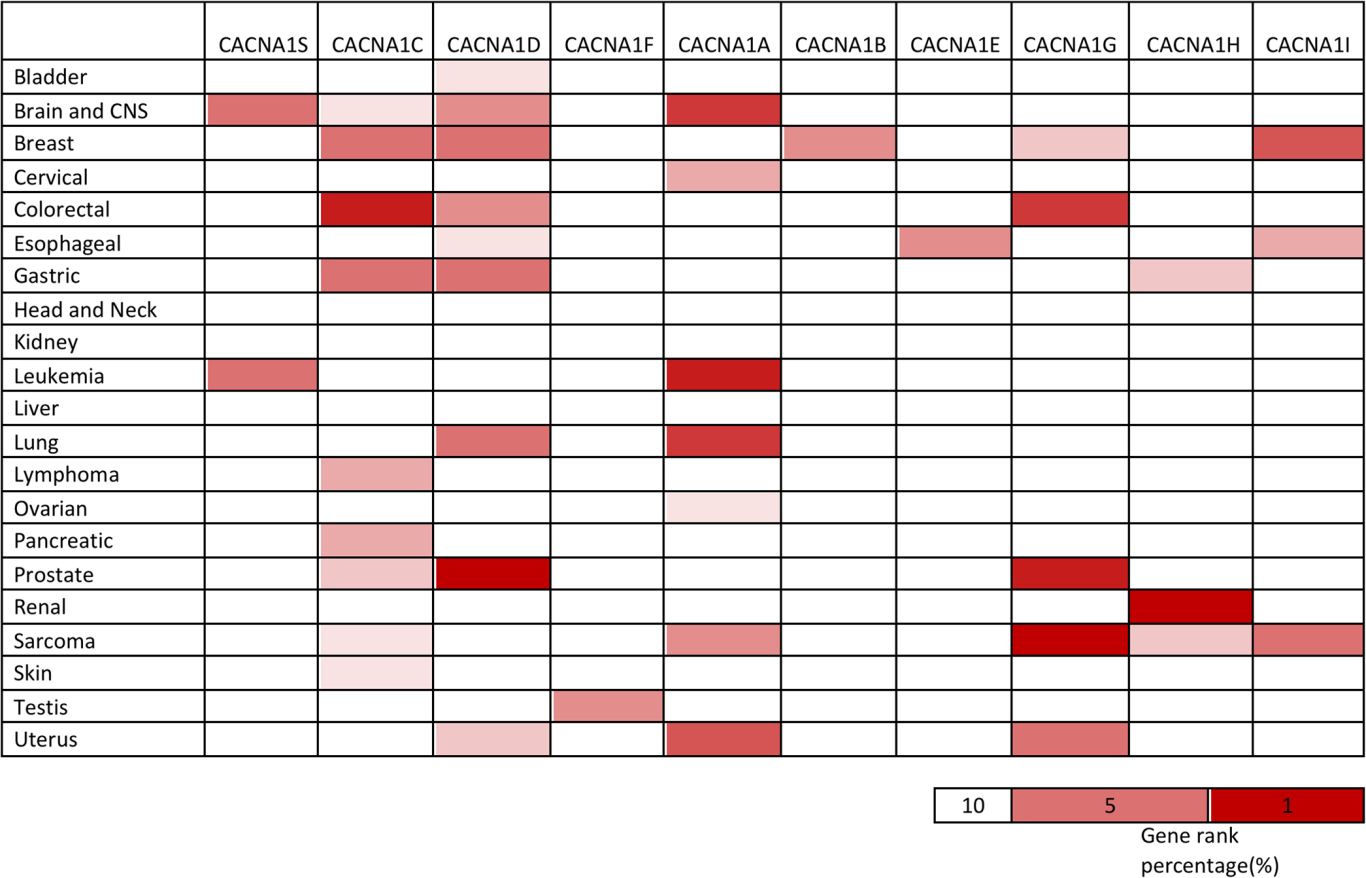
Expression of voltage-gated calcium channel (VGCC) genes in different types of cancer. Expression of voltage-gated calcium channel (VGCC) genes in 21 types of cancers compared to normal tissue controls. The gene name of each channel is shown. Each gene was found in its tissue of origin, and the color gradient correlates with decreasing gene rank percentile. The search criteria threshold was set at p-value<0.05 with fold change >1.5 and gene rank percentile <10% for screening microarray datasets of cancer versus normal cases.

The P/Q-type, T-type, N-type, R-type, and L type VGCCs all contain the α1 subunit responsible for assembling the calcium-selective pore [41, 69]. This subunit is encoded by various genes spreading from the L-type (CACNA1S, CACNA1C, CACNA1D and CACNA1F) to the T-type (CACNA1G, CACNA1H and CACNA1I) [70]. However, to date, no holistic approach has been taken to the screening VGCC family genes in different types of cancer. The present study used a holistic approach to explore VGCC expression in different types of cancer by employing the web-based ONCOMINE microarray database to analyze altered VGCC mRNA expression in 21 types of cancer. We compared the cancer tissue to normal tissue controls and set threshold criteria for screening a suitable dataset from the ONCOMINE database. Inclusion of a suitable dataset for further analysis required that comparisons of gene expression between cancer and normal tissues obeyed specific threshold criteria: the fold change must be above 1.5, the p-value must be less than 0.05, and the gene-ranking percentile must be less than 10%. The fold change, p-value, and the top gene-ranking percentile are presented in Fig 1 for different VGCC genes in different types of cancer tissues.

## 2. L-type calcium channel family

The L-type calcium channel genes investigated here include Cav1.1 (CACNA1S), Cav1.2 (CACNA1C), Cav1.3 (CACNA1D), and Cav1.4 (CACNA1F), commonly localized in smooth muscle, skeletal muscle, ventricular myocytes, and bone (osteoblasts). Previous studies on the role of the L-type calcium channel were primarily focused on the physiological and pharmacological aspects [71, 72]; hence, its function is largely unknown in terms of cancer diseases. Our data revealed that CACNA1S was overexpressed relative to normal tissue samples in acute myeloid leukemia (with a 2.42-fold change), in brain desmoplastic medulloblastoma (with a 1.89-fold change), and in primitive neuroectodermal tumors (with a 1.81-fold change) (Table 2). CACNA1S also ranked in the top 5% of upregulated genes in both leukemia and brain cancer (Fig 1).

**Table 2:** L-type calcium channel expression in cancer.

Gene	Cancer	Subtype	N (case)	P-value (Cancer/Normal)	t-Test (Cancer/Normal)	Fold (Cancer/Normal)	% Gene Ranking	Database References
CACNA1S	Brain	Desmoplastic Medulloblastoma	85	0.002	3.988	1.894	356 (in top 7%)	Nature 2002/01/24[5]
		Primitive Neuroectodermal Tumor, NOS	85	0.015	2.671	1.816	266 (in top 5%)	Nature 2002/01/24[5]
	Leukemia	Acute Myeloid Leukemia	87	0.005	3.121	2.427	578 (in top 5%)	Nat Genet 2004/03/01[6]
CACNA1C	Colorectal	Adenocarcinoma	105	7.33E-14	9.235	1.642	214 (in top 2%)	PLoS One 2010/10/01 [73]
		Colon Adenoma	64	4.88E-11	7.974	4.324	1145 (in top 6%)	Mol Cancer Res 2007/12/01[74]
		Rectal Adenoma	64	2.58E-5	5.831	3.795	1416 (in top 8%)	Mol Cancer Res 2007/12/01[74]
	Gastric	Gastrointestinal Stromal Tumor	90	1.34E-4	7.113	2.365	63 (in top 5%)	Clin Cancer Res 2011/04/01[75]
		Gastric Mixed Adenocarcinoma	69	4.47E-4	4.609	2.222	1289 (in top 7%)	Eur J Cancer 2009/02/01[76]
	Pancreas	Pancreatic Adenocarcinoma	27	4.07E-4	4.484	13.118	329 (in top 7%)	Cancer Res 2003/05/15[77]
	Sarcoma	Synovial Sarcoma	54	8.15E-4	3.899	2.365	1060 (in top 9%)	Cancer Res 2005/07/01[78]
	Leukemia	B-Cell Childhood Acute Lymphoblastic Leukemia	288	0.004	5.691	4.155	769 (in top 7%)	Blood 2011/06/09[79]
		Marginal Zone B-Cell Lymphoma	27	0.027	2.449	1.514	1254 (in top 9%)	
	Brain	Glioblastoma	101	0.006	5.655	8.620	1918 (in top 10%)	Cancer Cell 2006/05/01[80]
		Primitive Neuroectodermal Tumor	85	0.015	2.671	1.816	47 (in top 9%)	Nature 2002/01/24 [5]
		Oligodendroglioma	54	0.020	2.665	2.651	1342 (in top 10%)	Cancer Res 2005/10/01[81]
	Breast	Breast Phyllodes Tumor	2136	0.009	3.731	1.529	1310 (in top 7%)	Nature 2012/04/18[82]
		Invasive Lobular Breast Carcinoma	30	0.025	2.142	1.901	943 (in top 5%)	BMC Cancer 2007/03/27[83]
	Uterus	Uterine Corpus Leiomyosarcoma	24	0.017	2.430	1.509	10 (in top 10%)	Genes Chromosomes Cancer 2004/06/01[84]
	Skin	Skin Squamous Cell Carcinoma	15	0.018	2.673	2.767	1050 (in top 9%)	Mol Cancer 2006/08/08 [85]
	Prostate	Prostate Carcinoma	35	0.024	2.671	1.622	670 (in top 8%)	Cancer Res 2002/08/01[86]
CACNA1D	Prostate	Carcinoma	112	3.31E-11	7.543	2.138	113 (in top 2%)	PNAS 2004/01/20 [87]
		Carcinoma	122	4.17E-10	6.929	2.626	133 (in top 1%)	Nature 2012/05/20 [7]
		Carcinoma	185	5.13E-10	6.873	1.828	111 (in top 1%)	Cancer Cell 2010/07/13[88]
		Prostate Carcinoma Epithelia	101	7.70E-8	6.104	5.972	46 (in top 1%)	Nat Genet 2007/01/01[89]
		Prostatic Intraepithelial Neoplasia Epithelia	101	0.003	3.131	4.682	1060 (in top 10%)	Nat Genet 2007/01/01[89]
		Adenocarcinoma	40	2.42E-6	5.453	2.199	176 (in top 1%)	Cancer Res 2003/07/15[90]
		Carcinoma	57	1.53E-5	4.566	1.747	1 (in top 1%)	Cancer Res 2006/04/15[91]
		Carcinoma	21	2.57E-5	5.486	4.061	49 (in top 1%)	Clin Cancer Res 2009/09/15[92]
		Adenocarcinoma	89	3.57E-4	3.760	2.059	393 (in top 4%)	Cancer Res 2008/02/01[93]
		Carcinoma	30	0.002	3.439	6.348	127 (in top 1%)	Mol Carcinog 2002/01/01[94]
		Carcinoma	15	0.015	2.701	17.129	197 (in top 4%)	Cancer Res 2001/08/01[95]
	Breast	Invasive Lobular Breast Carcinoma	593	2.52E-10	7.399	3.431	1031 (in top 6%)	TCGA
		Mixed Lobular and Ductal Breast Carcinoma	593	1.35E-4	6.197	4.200	914 (in top 5%)	TCGA
		Invasive Ductal and Lobular Carcinoma	593	0.002	8.208	4.839	1474 (in top 8%)	TCGA
		Invasive Mixed Breast Carcinoma	63	0.011	2.804	4.365	708 (in top 5%)	PNAS 2005/08/02 [96]
		Invasive Ductal Breast Carcinoma	63	0.021	2.354	2.991	1157 (in top 7%)	PNAS 2005/08/02 [96]
		Invasive Lobular Breast Carcinoma	63	0.025	2.222	2.996	1025 (in top 7%)	PNAS 2005/08/02 [96]
	Colorectal	Adenocarcinoma	105	2.45E-8	6.148	1.527	1089 (in top 6%)	PLoS One 2010/10/01[73]
		Adenoma	105	1.32E-5	6.949	3.577	1150 (in top 6%)	PLoS One 2010/10/01[73]
		Rectosigmoid Adenocarcinoma	237	1.68E-5	5.628	1.788	663 (in top 4%)	TCGA
	Bladder	Superficial Bladder Cancer	60	4.49E-6	5.087	2.114	1089 (in top 9%)	Cancer Res 2004/06/01[97]
	Gastric	Gastric Mixed Adenocarcinoma	69	1.13E-4	5.235	3.467	856 (in top 5%)	Eur J Cancer 2009/02/01[76]
		Gastric Cancer	160	7.45E-4	3.246	1.519	1058 (in top 6%)	Nucleic Acids Res 2011/03/01[98]
	Lung	Lung Carcinoid Tumor	203	2.50E-4	4.121	3.611	396 (in top 5%)	PNAS 2001/11/20[99]
	Brain	Glioblastoma	101	3.85E-4	6.345	3.293	1069 (in top 6%)	Cancer Cell 2006/05/01[80]
	Uterus	Uterine Corpus Leiomyoma	77	5.44E-4	3.496	2.143	1492 (in top 8%)	Cancer Res 2009/08/01 [14]
	Esophagus	Adenocarcinoma	48	6.66E-4	4.155	2.447	318 (in top 9%)	Gastroenterology 2006/09/01[15]
		Barrett's Esophagus	48	0.002	3.242	2.123	1158 (in top 8%)	Gastroenterology 2006/09/01[15]
CACNA1F	Testis	Testicular Teratoma	30	0.018	2.859	1.896	829 (in top 6%)	Cancer Res 2005/07/01[78]

Previous research showed that CACNA1C could cause pathophysiology of psychiatric disease [100], and CACNA1C has high transcript activity in the prostate stroma [101]. We found high CACNA1C expression in prostate carcinoma in comparison to normal tissue in the Cancer research 2002/08/01 [86] database (Table 2). These data are consistent with those of a previous study [101]. We also found high expression of CACNA1C in most cancer types, including colorectal, gastric, pancreas, brain, breast, uterus, skin, and prostate cancers and leukemia (Table 2). We further found that 10 out of 21 different tumor tissues showed upregulation, with CACNA1C appearing in the top 10% of the most augmented genes (Fig 1). For example, colorectal cancers such as colon adenoma, adenocarcinoma, and rectal adenoma showed significant upregulation of CACNA1C when compared to normal control tissues, with p-values ranging from 2.58E-5 to 7.33E-14 and CACNA1C ranking from 2% to 8%. CACNA1C expression was also elevated in pancreatic carcinoma compared to normal tissue, with a 13.118-fold increase, a p-value of 4.07E-4, and gene ranking at 7%.

CACNA1D is believed to regulate cell firing [102] and has a high correlation with prostate cancer [17]; however, its expression in other cancer types is still largely unstudied. Our bioinformatics analysis verified that CACNA1D was highly expressed in most types of cancer, including prostate and breast cancer (Table 2). These data are consistent with the findings of a previous study [17]. We also found that 9 of the 21 tissue sections from cancer patients showed overexpression, with CACNA1D categorized in the top 10% of the most elevated genes (Fig 1). Prostate cancers such as prostate carcinoma, intraepithelial neoplasia, and adenocarcinoma all showed dramatic overexpression of CACNA1D relative to normal tissues. Upregulation ranged from 1.747- to 17.129-fold in terms of CACNA1D transcript expression, with p-values ranging from 0.015 to 3.31E-11 and gene rankings ranging from the top 1% to the top 4%. Breast cancers such as invasive lobular breast carcinoma, invasive ductal and lobular carcinoma, mixed lobular and ductal breast carcinoma, and invasive mixed breast carcinoma all exhibited substantial overexpression of CACNA1D relative to control samples. Upregulation ranged from 2.99- to 4.84-fold in terms of CACNA1D transcript expression, with p-values ranging from 0.025 to 2.52E-10 and gene rankings ranging from the top 5% to the top 7%. A particularly novel finding was that CACNA1D was highly expressed in prostate cancer but also in breast, colorectal, bladder, gastric, lung, brain, uterine, and esophageal tumors. Our *in silico* analysis suggests that CACNA1D may be a novel oncogene in cancer development, but further experiments are needed to explore the details of the role of CACNA1D in cancer progression.

A larger role in human physiology beyond its function in photoreceptors was suggested for CACNA1F [102]; however, the role of CACNA1F in cancer remains obscure. Only one study satisfied the selection benchmark with a 1.89-fold change in CACNA1F expression in testicular teratoma [103], wherein CACNA1F ranked in the top 6% of testicular teratoma gene changes and the p-value was 0.018 (Table 2).

### 3. P/Q-type calcium channel family

Cav2.1 (CACNA1A) is the only gene belonging to the P/Q-type calcium channel family, and it is often localized in Purkinje cells or cerebellar granule cells. This channel plays roles in neurotransmission and dendritic calcium transients [19]. P-type and Q-type currents are different in location. P-type are located in the Purkinje neurons of the cerebellum whereas Q-Type have been identified in cerebellar granule neurons [104, 105]. Both types of currents are produced by ion channels encoded by the calcium channel, voltage-dependent, P/Q type, alpha 1A subunit (CACNA1A) gene. They are phenotypically distinguished by an RNA splicing variation [106, 107]. Different mutations in alpha subunit 1A lead to certain neuronal degradation diseases such as episodic ataxia type-2, familial hemiplegic migraine and spinocerebellar ataxia type-6 [108–112]. In the present study, we found that CACNA1A was highly expressed in most cancers, including leukemia and ovarian cancer (Table 3). We also found that 7 out of 21 cancer tissues showed high expression of CACNA1A, and it was categorized in the top 10% of the most increased genes (Fig 1). Leukemias such as chronic lymphocytic leukemia, monoclonal gammopathy of undetermined significance, skin squamous cell carcinoma, and marginal zone b-cell lymphoma all presented significant overexpression of CACNA1A relative to control samples. The *in silico* analysis showed increased expression ranging from 1.77- to 2.27-fold for CACNA1A with p-values ranging from 0.003 to 3.56E-80 and gene rankings ranging from the top 2% to 5%. Lung carcinoma cells showed the most significant increases in expression relative to control samples with 15.568-fold up-regulation, a p-value of 0.001, and a gene ranking in the top 4%. Overall, our bioinformatics analysis indicated that CACNA1A may be a potential therapeutic target for leukemia, lung, ovarian, brain, uterine, and cervical cancers.

**Table 3:** P-type calcium channel expression in cancer.

Gene	Cancer	Subtype	N (case)	P-value (Cancer/Normal)	t-Test (Cancer/Normal)	Fold (Cancer/Normal)	% Gene Ranking	Database References
CACNA1A	Leukemia	Chronic Lymphocytic Leukemia	2096	3.56E-80	23.560	1.765	232 (in top 2%)	J Clin Oncol 2010/05/20 [114]
		Monoclonal Gammopathy of Undetermined Significance	78	1.33E-6	5.258	2.053	767 (in top 4%)	Blood 2007/02/15 [115]
		Skin Squamous Cell Carcinoma	87	2.31E-4	4.688	3.389	35 (in top 5%)	BMC Med Genomics 2008/04/28 [116]
		Marginal Zone B-Cell Lymphoma	27	0.003	3.787	2.271	189 (in top 2%)	J Invest Dermatol 2003/05/01 [8]
	Ovarian	Carcinoma	195	5.20E-8	8.750	1.758	1087 (in top 9%)	Cancer Res 2008/07/01 [9]
	Sarcoma	Myxoid/Round Cell Liposarcoma	158	8.07E-8	7.026	1.711	905 (in top 8%)	Nat Genet 2010/07/04 [10]
		Dedifferentiated Liposarcoma	158	1.86E-7	5.847	1.575	706 (in top 6%)	Nat Genet 2010/07/04 [10]
		Synovial Sarcoma	54	2.38E-4	4.461	4.470	712 (in top 6%)	Cancer Res 2005/07/01 [78]
	Brain	Classic Medulloblastoma	85	5.24E-6	4.935	6.574	305 (in top 6%)	Nature 2002/01/24 [5]
		Primitive Neuroectodermal Tumor, NOS	85	0.015	2.671	1.816	390 (in top 8%)	Nature 2002/01/24 [5]
		Glioblastoma	101	6.67E-6	6.947	5.843	550 (in top 3%)	Cancer Cell 2006/05/01 [80]
	Uterus	Uterine Corpus Leiomyoma	77	1.22E-5	4.578	2.687	602 (in top 4%)	Cancer Res 2009/08/01 [14]
	Ovaria	Ovarian Serous Cystadenocarcinoma	594	1.47E-5	8.013	2.563	1077 (in top 9%)	TCGA
	Lung	Lung Carcinoid Tumor	203	2.26E-5	4.656	6.098	222 (in top 3%)	PNAS 2001/11/20 [99]
		Small Cell Lung Carcinoma	203	0.001	4.583	15.568	320 (in top 4%)	PNAS 2001/11/20 [99]
	Cervix	High Grade Cervical Squamous Intraepithelial Neoplasia Epithelia	41	0.004	3.541	1.601	873 (in top 7%)	Cancer Res 2007/11/01 [117]

When applying Kaplan-Meier plotter analysis, correlations between the overexpression of CACNA1A and overall lower survival rates in lung cancer [53] and ovarian cancer (S2 Fig) were shown by using the GSE9891 database [51, 113]. This result is consistent with our data in Table 3. The high expression of CACNA1A shows that this gene is possibly involved in the onset and progression of lung and ovarian cancer (poor prognosis). In contrast, an opposite trend was observed in breast and stage IV gastric cancer with low expression of CACNA1A [52]. These data show consistency with Fig 1. In other words, CACNA1A was down-regulated in breast and gastric cancer (S3 Fig). These studies showed that CACNA1 expression plays an essential role in the progression of ovarian and lung cancer.

### 4. N-type calcium channel family

The N-type calcium channel family contains only Cav2.2 (CACNA1B), which is located throughout the brain and peripheral nervous system. Previous studies have shown that CACNA1B is important for sustained neuronal firing and neurotransmitter release in neuropathic pain [25, 29]; however, until now, CACNA1B has not been implicated in cancer. Our bioinformatics results indicated thatCACNA1B was among the top 9% and top 6% of overexpressed genes in prostate and breast cancer, respectively. In these cancers, increases in CACNA1B expression ranged from 1.53- to 1.56-fold, with p-values from 3.25E-4 to 6.22E-4 relative to control samples (Table 4). Our data suggest that CACNA1B has high expression specifically in clinical prostate and breast cancer tissues. Identification of the underlying role of CACNA1B in cancer development may also help in the discovery of new therapeutic targets for the treatment of prostate and breast cancer.

**Table 4:** N-type calcium channel expression in cancer.

Gene	Cancer	Subtype	N (case)	P-value (Cancer/Normal)	t-Test (Cancer/Normal)	Fold (Cancer/Normal)	% Gene Ranking	Database References
CACNA1B	Prostate	Carcinoma	122	3.25E-4	3.624	1.532	1710 (in top 9%)	Nature 2012/05/20 [7]
	Breast	Intraductal Cribriform Breast Adenocarcinoma	593	6.22E-4	3.418	1.564	1032 (in top 6%)	TCGA

### 5. T-type calcium channel family

Cav3.1 (CACNA1G), Cav3.2 (CACNA1H), and Cav3.3 (CACNA1I) are all classified into the T-type calcium channel family, which is localized in neuronal cells, pacemaker cells and osteocytes (mature bone cells). In addition, another study using ONCOMINE showed that the expression of T-type channel isoforms in an array of malignant tumor cells was significantly elevated relative to surrounding normal tissue [118]. This outcome is consistent with the present findings (Table 5). Increased expression of CACNA1G was detected in a broad range of cancer diseases, with CACNA1G in the top 1% of overexpressed genes in synovial sarcoma and in the top 2% in prostate carcinoma. The fold changes ranged from 1.737 to 6.376 and the p-values from 8.70E-4 to 1.71E-7 (Table 5). High expression of CACNA1G was also noted in other tumor types such as colorectal, uterine, prostate, and breast cancer.

**Table 5:** T-type calcium channel expression in cancer.

Gene	Cancer	Subtype	N (case)	P-value (Cancer/Normal)	t-Test (Cancer/Normal)	Fold (Cancer/Normal)	% Gene Ranking	Database References
CACNA1G	Sarcoma	Synovial Sarcoma	54	1.71E-7	9.065	6.376	42 (in top 1%)	Cancer Res 2005/07/01 [11]
		Dedifferentiated Liposarcoma	54	0.002	3.374	1.850	332 (in top 3%)	Cancer Res 2005/07/01 [11]
	Colorectal	Rectosigmoid Adenocarcinoma	237	3.72E-6	5.749	1.866	516 (in top 3%)	TCGA
	Uterus	Uterine Corpus Leiomyoma	77	4.21E-5	4.279	1.743	796 (in top 5%)	Cancer Res 2009/08/01 [14]
	Lung	Adenocarcinoma	66	7.72E-4	3.334	1.956	215 (in top 10%)	BMC Genomics 2007/06/01 [119]
	Prostate	Carcinoma	19	8.70E-4	4.132	1.737	302 (in top 2%)	Cancer Cell 2005/11/01 [120]
	Breast	Invasive Lobular Breast Carcinoma	30	0.042	1.908	2.007	1533 (in top 8%)	BMC Cancer 2007/03/27 [83]
CACNA1H	Renal	Clear Cell Sarcoma of the Kidney	35	1.51E-6	7.591	5.193	112 (in top 1%)	Clin Cancer Res 2005/11/15 [12]
		Renal Wilms Tumor	35	0.005	3.566	1.704	808 (in top 7%)	Clin Cancer Res 2005/11/15 [12]
	Sarcoma	Synovial Sarcoma	54	5.68E-4	4.402	6.103	940 (in top 8%)	Cancer Res 2005/07/01 [78]
	Gastric	Gastrointestinal Stromal Tumor	90	5.69E-4	6.075	9.290	1509 (in top 8%)	Clin Cancer Res 2011/04/01 [75]
CACNA1I	Breast	Invasive Breast Carcinoma Stroma	59	3.04E-16	15.313	2.348	758 (in top 4%)	Nat Med 2008/05/01 [13]
		Ductal Breast Carcinoma in Situ Epithelia	66	0.002	3.748	1.566	1241 (in top 7%)	Breast Cancer Res 2009/02/02
	Sarcoma	Myxoid/Round Cell Liposarcoma	158	9.11E-9	7.885	1.899	628 (in top 5%)	Nat Genet 2010/07/04 [10]
	Esophagus	Esophageal Adenocarcinoma	48	3.43E-4	5.451	2.436	1014 (in top 7%)	Gastroenerology 2006/09/01[15]

CACNA1H showed altered expression in renal cancer, sarcoma, and gastrointestinal stromal tumors (Fig 1). CACNA1H was located in the top 1% of overexpressed genes in clear cell sarcoma of the kidney and in the top 8% of upregulated genes in synovial sarcoma and gastrointestinal stromal tumors. Compared to normal tissue, the fold change ranged from 5.19 to 9.29 and p-values ranged from 1.51E-6 to 0.005.

CACNA1I showed altered expression in invasive breast cancer, myxoid/round cell liposarcoma, and esophageal adenocarcinoma (Fig 1). CACNA1I was found in the top 4% to 7% of upregulated genes in invasive breast carcinoma stroma and ductal breast carcinoma *in situ* epithelia, with p-values of 3.04E-16 and 0.002 and fold changes ranging from 1.586 to 2.35, respectively. High expression of CACNA1I was also found in sarcoma and esophageal cancer (Table 5).

T-type calcium channels have recently drawn the attention of researchers as potential therapeutic targets in cancer treatment. T-type channels are well documented to be involved in cell growth and differentiation, to be re-expressed in various tumor phases, and to be involved in calcium-mediated cell death. T-type calcium channels are highly expressed in most types of cancer [121, 122]. Therefore, the development of a specific inhibitor or antagonist drug may serve as a potential approach to treating cancer.

### 6. R-type calcium channel family

The R-type calcium channel family contains only Cav2.3 (CACNA1E), which is most often found in cerebellar granule cells and other neurons. CACNA1E was among the top 6% and top 10% of genes overexpressed in esophageal and uterine cancers, respectively. In those cancers, CACNA1E expression increases ranged from 2.09- to 9.19-fold, with p-values from 1.91E-4 to 0.001 relative to the control samples (Table 6). Hence, CACNA1E may also serve as a novel therapeutic target for esophageal and uterine cancers.

**Table 6:** R-type calcium channel expression in cancer.

Gene	Cancer	Subtype	N (case)	P-value (Cancer/Normal)	t-Test (Cancer/Normal)	Fold (Cancer/Normal)	% Gene Ranking	Database References
CACNA1E	Esophagus	Adenocarcinoma	48	1.91E-4	5.855	9.193	829 (in top 6%)	Gastroenterology 2006/09/01[15]
	Uterus	Uterine Corpus Leiomyoma	77	0.001	3.148	2.095	1901 (in top 10%)	Cancer Res 2009/08/01 [14]

### 7. VGCCs and their relationship to metastatic cancer

Cancer cells are able to metastasize or spread to other tissues or organs during tumor growth [123]. As the original tumor progresses through angiogenesis [124], it supposedly promotes the circulation of cancerous cells in the peripheral blood system [125] or lymphatic system [126] and their migration to other tissues or organs [127]. These cells then begin growing in the host organs. However, these metastatic growths are not easy to detect and often lead to the death of the patient. Gene expression profiling of human primary breast tumors can predict metastasis risk, and metastatic cancer is also often correlated with poor prognosis [128]. Therefore, understanding the association between VGCCs and metastatic cancer represents an important facet of cancer research. However, the correlation between VGCCs and metastatic cancer remains obscure. Hence, exploration of the VGCC gene expression profiles in clinical cancer patients may be useful for predicting metastasis risk.

Invasive lobular breast carcinoma has been frequently found to metastasize to the gastrointestinal tract, peritoneum, retroperitoneum, and gynecological organs [129–131]. The BMC Cancer database [83] revealed CACNA1C expression in invasive lobular breast carcinoma/normal tissue with a 1.9-fold change (Table 2); thus, we speculated that patients with invasive lobular breast carcinoma with high expression of CACNA1C relative to normal tissue were at risk for metastasis to the gastrointestinal tract, peritoneum, retroperitoneum, and gynecological organs.

The TCGA and PNAS databases [96] indicated that CACNA1D was significantly overexpressed relative to normal tissue in invasive lobular breast carcinoma with invasive ductal and lobular carcinoma (Table 2), which again implies that patients with high expression of CACNA1D were likely to develop those diseases.

The BMC Cancer database [83] revealed that CACNA1G expression in invasive lobular breast carcinoma samples underwent a 2.0-fold change relative to normal samples (Table 5). This also implies that patients with CACNA1G overexpression relative to normal tissue were likely to experience gastrointestinal tract, peritoneum, retroperitoneum, or gynecological organ transfer. In addition, abundant expression of JMJD2C was noted in invasive breast carcinoma stroma, which would also lead to metastatic disease [132]. The Nat Med database [13] showed a 2.3-fold change in CACNA1I in invasive breast carcinoma stroma, again implying that patients with high CACNA1G expression would likely develop cancer.

Most types of cancer, including blood cancers and lymphatic system cancers (i.e., leukemia, multiple myeloma, and lymphoma), are able to bring about metastatic tumors. Although rare, blood and lymphatic system cancers have been reported to metastasize to other organs such as the lungs, heart, central nervous system, and other tissues [133–136]. Cardiac metastases were found in 53 out of 247 necropsied patients with leukemia or lymphoma [137]. The Nat Genet database indicated that L-type calcium channels, such as CACNA1S, were overexpressed in leukemia/normal tissue with a 2.42-fold change [6] (Table 2). The Blood database indicated high expression of CACNA1C in leukemia relative to normal samples [79] (Table 2). We also found that the P-type CACNA1A calcium channel gene was highly expressed in leukemia compared to normal samples [8, 9, 115, 116] (Table 3). Thus, we speculated that leukemia patients with high expression of CACNA1S, CACNA1C, or CACNA1A relative to normal samples are likely to experience metastasis of the cancer cells to the lungs, heart, central nervous system, and other tissues.

### 8. Voltage-gated calcium channels in clinical applications

*In silico* bioinformatics analysis is playing an important role in linking cancer gene expression profiling with potential clinical cancer markers. This type of systematic analysis provides a holistic global view of the clinical data for VGCC gene family expression in various types of cancer diseases, and it also confirmed that expression of VGCC genes may change greatly in metastatic diseases. One interesting feature was that various types of VGCC genes appear to take part in diverse types of cancer. For instance, breast cancer showed dramatic upregulation of CACNA1C, CACNA1D, CACNA1B, CACNA1G, and CACNA1I [13, 82, 83, 96, 138]. Likewise, brain and CNS tumors showed significantly increased expression of CACNA1S, CACNA1C, CACNA1D, and CACNA1A [5, 80]. Our results indicate that CACNA1F is highly expressed only in testis cancer and that CACNA1B is up-regulated only in breast cancer.

Our approach to bioinformatics analysis also utilized the integration and validation of multiple microarray datasets so that the most novel voltage-gated calcium channel markers could be identified for further investigation. Identifying novel VGCC targets and classifying different subtypes of cancers on the basis of DNA microarray data may promote the development of new cancer therapy drugs.

Recently, overexpression of the L-type CACNA1D calcium channel gene was confirmed in prostate cancer [139]. In the current research, the CACNA1 family was found to be highly expressed in several varieties of cancer including breast, bladder, colorectal, lung, esophageal, brain and CNS, uterine, and gastric cancers. The finding of an association between colorectal cancer and CACNA1D strongly suggests a new direction for cancer diagnosis and treatment. CACNA1D was found to be expressed in colorectal cancer in the 6th percentile in terms of gene ranking (from the 1st to 10th percentile).

Some studies on calcium channel blockers have been conducted to identify potential targets for cancer suppression [140, 141]. Ligand-gated calcium channels have also been identified as potential therapeutic targets apart from VGCCs. A recent study indicated an association between oncogenic K-Ras IP3-dependent suppression and a calcium release mechanism that strongly suggests a role for IP3 in the function of ligand-gated calcium channels involved in colorectal cancer [142].

In conclusion, the current findings show the overexpression of calcium channels in a number of cancer diseases. The overexpression of many calcium channel subunits in cancers shows that they are likely involved in the development of various types of cancer. The observation of overexpression of CACNA1A, CACNA1C, and CACNA1D could make them likely targets in cancer treatment, as it suggests that blockage or partial inhibition of their expression could help to modulate the status of metastatic diseases. However, further detailed investigations on the mechanism of how calcium channel subunits play roles in cancer onset and progression need to be conducted. The present study could serve as a tool for cancer diagnostics and assist in the search more applicable and specific types of cancer treatments.

## Supporting Information

S1 Fig Flow chart presenting the identification and collection of the studies for the statistical meta-analysis. (TIF)

S2 Fig The CACNA1A gene in breast, gastric, ovarian and lung cancer (Kaplan-Meier Plotter). Kaplan-Meier plots showing overall survival in breast, gastric, ovarian and lung cancer. Over-expression of CACNA1A in ovarian and lung cancer would cause poor prognosis, whereas in breast and gastric it would lead to good prognosis. Breast cancer, p = 1.4X 10–7; gastric cancer, p = 0.038; ovarian cancer, p = 0.001; lung cancer, p = 2.4 X10-5.
(TIF)

S3 Fig CACNA1A gene analysis in breast, gastric, ovarian and lung cancer (ONCOMINE database). Box plots derived from gene expression data in ONCOMINE comparing expression of the CACNA1A gene in normal (left plot) and various types of cancer tissue (right plot).
(TIF)

S1 Table PRISMA 2009 Checklist. (DOCX)

S2 Table ONCOMINE dataset reference list. (DOCX)

## References

[1] Prevarskaya N, Skryma R, Shuba Y. Ion channels and the hallmarks of cancer. Trends in molecular medicine. 2010;16(3):107–21. 10.1016/j.molmed.2010.01.005 20167536

[2] Lipskaia L, Lompré AM. Alteration in temporal kinetics of Ca2+ signaling and control of growth and proliferation. Biology of the Cell. 2004;96(1):55–68. 15093128 10.1016/j.biolcel.2003.11.001

[3] Schreiber R. Ca2+ signaling, intracellular pH and cell volume in cell proliferation. The Journal of membrane biology. 2005;205(3):129–37. 16362501 10.1007/s00232-005-0778-z

[4] Panner A, Wurster RD. T-type calcium channels and tumor proliferation. Cell calcium. 2006;40(2):253–9. 16765439 10.1016/j.ceca.2006.04.029

[5] Pomeroy SL, Tamayo P, Gaasenbeek M, Sturla LM, Angelo M, McLaughlin ME, et al Prediction of central nervous system embryonal tumour outcome based on gene expression. Nature. 2002;415(6870):436–42. 11807556 10.1038/415436a

[6] Stegmaier K, Ross KN, Colavito SA, O'Malley S, Stockwell BR, Golub TR. Gene expression–based high-throughput screening (GE-HTS) and application to leukemia differentiation. Nature genetics. 2004;36(3):257–63. 14770183 10.1038/ng1305

[7] Grasso CS, Wu Y-M, Robinson DR, Cao X, Dhanasekaran SM, Khan AP, et al The mutational landscape of lethal castration-resistant prostate cancer. Nature. 2012;487(7406):239–43. 10.1038/nature11125 22722839 PMC3396711

[8] Storz MN, van de Rijn M, Kim YH, Mraz-Gernhard S, Hoppe RT, Kohler S. Gene expression profiles of cutaneous B cell lymphoma. J Invest Dermatol. 2003;120(5):865–70. 10.1046/j.1523-1747.2003.12142.x . 12713594

[9] Bonome T, Levine DA, Shih J, Randonovich M, Pise-Masison CA, Bogomolniy F, et al A gene signature predicting for survival in suboptimally debulked patients with ovarian cancer. Cancer research. 2008;68(13):5478–86. 10.1158/0008-5472.CAN-07-6595 18593951 PMC7039050

[10] Barretina J, Taylor BS, Banerji S, Ramos AH, Lagos-Quintana M, DeCarolis PL, et al Subtype-specific genomic alterations define new targets for soft-tissue sarcoma therapy. Nature genetics. 2010;42(8):715–21. 10.1038/ng.619 20601955 PMC2911503

[11] Skotheim RI, Lind GE, Monni O, Nesland JM, Abeler VM, Fosså SD, et al Differentiation of human embryonal carcinomas in vitro and in vivo reveals expression profiles relevant to normal development. Cancer research. 2005;65(13):5588–98. 15994931 10.1158/0008-5472.CAN-05-0153

[12] Cutcliffe C, Kersey D, Huang C-C, Zeng Y, Walterhouse D, Perlman EJ. Clear cell sarcoma of the kidney: up-regulation of neural markers with activation of the sonic hedgehog and Akt pathways. Clinical cancer research. 2005;11(22):7986–94. 16299227 10.1158/1078-0432.CCR-05-1354

[13] Finak G, Bertos N, Pepin F, Sadekova S, Souleimanova M, Zhao H, et al Stromal gene expression predicts clinical outcome in breast cancer. Nature medicine. 2008;14(5):518–27. 10.1038/nm1764 18438415

[14] Crabtree JS, Jelinsky SA, Harris HA, Choe SE, Cotreau MM, Kimberland ML, et al Comparison of human and rat uterine leiomyomata: identification of a dysregulated mammalian target of rapamycin pathway. Cancer research. 2009;69(15):6171–8. 10.1158/0008-5472.CAN-08-4471 19622772

[15] Hao Y, Triadafilopoulos G, Sahbaie P, Young HS, Omary MB, Lowe AW. Gene expression profiling reveals stromal genes expressed in common between Barrett’s esophagus and adenocarcinoma. Gastroenterology. 2006;131(3):925–33. 16952561 10.1053/j.gastro.2006.04.026 PMC2575112

[16] Szatkowski C, Parys JB, Ouadid-Ahidouch H, Matifat F. Inositol 1,4,5-trisphosphate-induced Ca(2+) signalling is involved in estradiol-induced breast cancer epithelial cell growth. Molecular Cancer. 2010;9:156 10.1186/1476-4598-9-156 PMC2906470. 20565939 PMC2906470

[17] Mariot P, Prevarskaya N, Roudbaraki MM, Le Bourhis X, Van Coppenolle F, Vanoverberghe K, et al Evidence of functional ryanodine receptor involved in apoptosis of prostate cancer (LNCaP) cells. The Prostate. 2000;43(3):205–14. 10797495 10.1002/(sici)1097-0045(20000515)43:3<205::aid-pros6>3.0.co;2-m

[18] Catterall WA, Perez-Reyes E, Snutch TP, Striessnig J. International Union of Pharmacology. XLVIII. Nomenclature and structure-function relationships of voltage-gated calcium channels. Pharmacological reviews. 2005;57(4):411–25. 16382099 10.1124/pr.57.4.5

[19] Catterall WA, Striessnig J, Snutch TP, Perez-Reyes E. International Union of Pharmacology. XL. Compendium of voltage-gated ion channels: calcium channels. Pharmacological Reviews. 2003;55(4):579–81. 14657414 10.1124/pr.55.4.8

[20] Tang S, Yatan A, Bahinski A, Mori Y, Schwartz A. Molecular localization of regions in the L-type calcium channel critical for dihydropyridine action. Neuron. 1993;11(6):1013–21. 8274273 10.1016/0896-6273(93)90215-d

[21] Bers DM. Cardiac excitation–contraction coupling. Nature. 2002;415(6868):198–205. 11805843 10.1038/415198a

[22] Tanabe T, Beam KG, Adams BA, Niidome T, Numa S. Regions of the skeletal muscle dihydropyridine receptor critical for excitation–contraction coupling. Nature. 1990;346(6284):567–9. 2165570 10.1038/346567a0

[23] Gomez A, Valdivia H, Cheng H, Lederer MR, Santana L, Cannell M, et al Defective excitation-contraction coupling in experimental cardiac hypertrophy and heart failure. Science. 1997;276(5313):800–6. 9115206 10.1126/science.276.5313.800

[24] Olivera BM, Miljanich G, Ramachandran J, Adams ME. Calcium channel diversity and neurotransmitter release: the ω-conotoxins and ω-agatoxins. Annual review of biochemistry. 1994;63(1):823–67. 10.1146/annurev.bi.63.070194.004135 7979255

[25] Uchitel O, Protti D, Sanchez V, Cherksey B, Sugimori M, Llinas R. P-type voltage-dependent calcium channel mediates presynaptic calcium influx and transmitter release in mammalian synapses. Proceedings of the National Academy of Sciences. 1992;89(8):3330–3. 10.1073/pnas.89.8.3330 PMC48860 1348859

[26] Ayata C, Shimizu-Sasamata M, Lo E, Noebels J, Moskowitz M. Impaired neurotransmitter release and elevated threshold for cortical spreading depression in mice with mutations in the α1A subunit of P/Q type calcium channels. Neuroscience. 1999;95(3):639–45. 10.1016/s0306-4522(99)00446-7 10670432

[27] Codignola A, Tarroni P, Clementi F, Pollo A, LoVallo M, Carbone E, et al Calcium channel subtypes controlling serotonin release from human small cell lung carcinoma cell lines. Journal of Biological Chemistry. 1993;268(35):26240–7. 8253745

[28] Mermelstein PG, Becker JB, Surmeier DJ. Estradiol reduces calcium currents in rat neostriatal neurons via a membrane receptor. Journal of Neuroscience. 1996;16(2):595–604. 8551343 10.1523/JNEUROSCI.16-02-00595.1996 PMC6578633

[29] Catterall WA. Structure and function of neuronal Ca2+ channels and their role in neurotransmitter release. Cell Calcium. 1998;24(5–6):307–23. doi: 10.1016/S0143-4160(98)90055-0. 10091001

[30] Boland LM, Bean BP. Modulation of N-type calcium channels in bullfrog sympathetic neurons by luteinizing hormone-releasing hormone: kinetics and voltage dependence. The Journal of neuroscience. 1993;13(2):516–33. 7678856 10.1523/JNEUROSCI.13-02-00516.1993 PMC6576628

[31] Westenbroek RE, Hell JW, Warner C, Dubel SJ, Snutch TP, Catterall WA. Biochemical properties and subcellular distribution of an N-type calcium hannel α1 subunit. Neuron. 1992;9(6):1099–115. 1334419 10.1016/0896-6273(92)90069-p

[32] Mackie K, Hille B. Cannabinoids inhibit N-type calcium channels in neuroblastoma-glioma cells. Proceedings of the National Academy of Sciences. 1992;89(9):3825–9. 10.1073/pnas.89.9.3825 PMC525583 1315042

[33] Diverse-Pierluissi M, Goldsmith PK, Dunlap K. Transmitter-mediated inhibition of N-type calcium channels in sensory neurons involves multiple GTP-binding proteins and subunits. Neuron. 1995;14(1):191–200. 7826637 10.1016/0896-6273(95)90254-6

[34] Markram H, Helm PJ, Sakmann B. Dendritic calcium transients evoked by single back-propagating action potentials in rat neocortical pyramidal neurons. The Journal of physiology. 1995;485(Pt 1):1–20. 7658365 10.1113/jphysiol.1995.sp020708 PMC1157968

[35] Yasuda R, Sabatini BL, Svoboda K. Plasticity of calcium channels in dendritic spines. Nature neuroscience. 2003;6(9):948–55. 12937422 10.1038/nn1112

[36] Johnston D, Magee JC, Colbert CM, Christie BR. Active properties of neuronal dendrites. Annual review of neuroscience. 1996;19(1):165–86. 10.1146/annurev.ne.19.030196.001121 8833440

[37] Carlin K, Jones K, Jiang Z, Jordan L, Brownstone R. Dendritic L‐type calcium currents in mouse spinal motoneurons: implications for bistability. European Journal of Neuroscience. 2000;12(5):1635–46. 10792441 10.1046/j.1460-9568.2000.00055.x

[38] Perez-Reyes E. Molecular physiology of low-voltage-activated t-type calcium channels. Physiological Reviews. 2003;83(1):117–61. 12506128 10.1152/physrev.00018.2002

[39] Bohn G, Moosmang S, Conrad H, Ludwig A, Hofmann F, Klugbauer N. Expression of T-and L-type calcium channel mRNA in murine sinoatrial node. FEBS letters. 2000;481(1):73–6. 10984618 10.1016/s0014-5793(00)01979-7

[40] Cribbs LL, Lee J-H, Yang J, Satin J, Zhang Y, Daud A, et al Cloning and characterization of α1H from human heart, a member of the T-type Ca2+ channel gene family. Circulation Research. 1998;83(1):103–9. 9670923 10.1161/01.res.83.1.103

[41] Ertel EA, Campbell KP, Harpold MM, Hofmann F, Mori Y, Perez-Reyes E, et al Nomenclature of voltage-gated calcium channels. Neuron. 2000;25(3):533–5. 10774722 10.1016/s0896-6273(00)81057-0

[42] Huguenard J, Prince D. A novel T-type current underlies prolonged Ca (2+)-dependent burst firing in GABAergic neurons of rat thalamic reticular nucleus. The Journal of neuroscience. 1992;12(10):3804–17. 1403085 10.1523/JNEUROSCI.12-10-03804.1992 PMC6575965

[43] Kito M, Maehara M, Watanabe K. Mechanisms of T-type calcium channel blockade by zonisamide. Seizure. 1996;5(2):115–9. 8795126 10.1016/s1059-1311(96)80104-x

[44] Hoheisel JD. Microarray technology: beyond transcript profiling and genotype analysis. Nature reviews genetics. 2006;7(3):200–10. 16485019 10.1038/nrg1809

[45] Rhodes DR, Kalyana-Sundaram S, Mahavisno V, Varambally R, Yu J, Briggs BB, et al Oncomine 3.0: genes, pathways, and networks in a collection of 18,000 cancer gene expression profiles. Neoplasia. 2007;9(2):166–80. 17356713 10.1593/neo.07112 PMC1813932

[46] Rhodes DR, Yu J, Shanker K, Deshpande N, Varambally R, Ghosh D, et al ONCOMINE: A Cancer Microarray Database and Integrated Data-Mining Platform. Neoplasia. 2004;6(1):1–6. doi: 10.1016/S1476-5586(04)80047-2. 15068665 PMC1635162

[47] Ewald JA, Downs TM, Cetnar JP, Ricke WA. Expression microarray meta-analysis identifies genes associated with Ras/MAPK and related pathways in progression of muscle-invasive bladder transition cell carcinoma. PloS one. 2013;8(2):e55414 10.1371/journal.pone.0055414 23383328 PMC3562183

[48] Moher D, Liberati A, Tetzlaff J, Altman DG. Preferred reporting items for systematic reviews and meta-analyses: the PRISMA statement. Annals of internal medicine. 2009;151(4):264–9. 19622511 10.7326/0003-4819-151-4-200908180-00135

[49] Ni M, Chen Y, Lim E, Wimberly H, Bailey ST, Imai Y, et al Targeting androgen receptor in estrogen receptor-negative breast cancer. Cancer cell. 2011;20(1):119–31. 10.1016/j.ccr.2011.05.026 21741601 PMC3180861

[50] D'Ambrogio A, Nagaoka K, Richter JD. Translational control of cell growth and malignancy by the CPEBs. Nature Reviews Cancer. 2013;13(4):283–90. 10.1038/nrc3485 23446545

[51] Gyorffy B, Lanczky A, Szallasi Z. Implementing an online tool for genome-wide validation of survival-associated biomarkers in ovarian-cancer using microarray data from 1287 patients. Endocr-Relat Cancer. 2012;19(2):197–208. 10.1530/Erc-11-0329 . 22277193

[52] Gyorffy B, Benke Z, Lanczky A, Balazs B, Szallasi Z, Timar J, et al RecurrenceOnline: an online analysis tool to determine breast cancer recurrence and hormone receptor status using microarray data. Breast Cancer Res Tr. 2012;132(3):1025–34. 10.1007/s10549-011-1676-y . 21773767

[53] Gyorffy B, Surowiak P, Budczies J, Lanczky A. Online Survival Analysis Software to Assess the Prognostic Value of Biomarkers Using Transcriptomic Data in Non-Small-Cell Lung Cancer. Plos One. 2013;8(12). doi: UNSP e82241 10.1371/journal.pone.0082241 . PMC3867325 24367507

[54] Berridge MJ. Calcium signalling and cell proliferation. Bioessays. 1995;17(6):491–500. 7575490 10.1002/bies.950170605

[55] Dziegielewska B, Brautigan DL, Larner JM, Dziegielewski J. T-Type Ca2+ Channel Inhibition Induces p53-Dependent Cell Growth Arrest and Apoptosis through Activation of p38-MAPK in Colon Cancer Cells. Molecular Cancer Research. 2014;12(3):348–58. 10.1158/1541-7786.MCR-13-0485 24362252

[56] Lee JY, Park SJ, Park SJ, Lee MJ, Rhim H, Seo SH, et al Growth inhibition of human cancer cells in vitro by T-type calcium channel blockers. Bioorganic & medicinal chemistry letters. 2006;16(19):5014–7. 16876410 10.1016/j.bmcl.2006.07.046

[57] Triggle DJ. Calcium channel antagonists: clinical uses—past, present and future. Biochemical pharmacology. 2007;74(1):1–9. 17276408 10.1016/j.bcp.2007.01.016

[58] Dziegielewska B, Gray LS, Dziegielewski J. T-type calcium channels blockers as new tools in cancer therapies. Pflügers Archiv-European Journal of Physiology. 2014;466(4):801–10. 10.1007/s00424-014-1444-z 24449277

[59] Taylor JT, Zeng X-B, Pottle JE, Lee K, Wang AR, Yi SG, et al Calcium signaling and T-type calcium channels in cancer cell cycling. World journal of gastroenterology: WJG. 2008;14(32):4984 18763278 10.3748/wjg.14.4984 PMC2742923

[60] Lee KS, Tsien RW. Mechanism of calcium channel blockade by verapamil, D600, diltiazem and nitrendipine in single dialysed heart cells. Nature. 1983;302(5911):790–4. 6302512 10.1038/302790a0

[61] Weiss J, Hartley D, Koh J-Y, Choi D. The calcium channel blocker nifedipine attenuates slow excitatory amino acid neurotoxicity. Science(Washington). 1990;247(4949):1474–7. 2157282 10.1126/science.247.4949.1474

[62] Haverstick DM, Heady TN, Macdonald TL, Gray LS. Inhibition of human prostate cancer proliferation in vitro and in a mouse model by a compound synthesized to block Ca2+ entry. Cancer research. 2000;60(4):1002–8. 10706116

[63] Enfissi A, Prigent S, Colosetti P, Capiod T. The blocking of capacitative calcium entry by 2-aminoethyl diphenylborate (2-APB) and carboxyamidotriazole (CAI) inhibits proliferation in Hep G2 and Huh-7 human hepatoma cells. Cell calcium. 2004;36(6):459–67. 15488595 10.1016/j.ceca.2004.04.004

[64] Chung SC, McDonald TV, Gardner P. Inhibition by SK&F 96365 of Ca2+ current, IL‐2 production and activation in T lymphocytes. British journal of pharmacology. 1994;113(3):861–8. 7858878 10.1111/j.1476-5381.1994.tb17072.x PMC1510420

[65] Azimi I, Roberts‐Thomson S, Monteith G. Calcium influx pathways in breast cancer: opportunities for pharmacological intervention. British journal of pharmacology. 2014;171(4):945–60. 10.1111/bph.12486 24460676 PMC3925034

[66] Loughlin KR. Calcium channel blockers and prostate cancer. Urologic Oncology: Seminars and Original Investigations. 2014;32(5):537–8. doi: 10.1016/j.urolonc.2013.08.001. 10.1016/j.urolonc.2013.08.001 24814406

[67] Zhuang L, Peng J-B, Tou L, Takanaga H, Adam RM, Hediger MA, et al Calcium-selective ion channel, CaT1, is apically localized in gastrointestinal tract epithelia and is aberrantly expressed in human malignancies. Laboratory investigation. 2002;82(12):1755–64. 12480925 10.1097/01.lab.0000043910.41414.e7

[68] Hall DD, Wu Y, Domann FE, Spitz DR, Anderson ME. Mitochondrial Calcium Uniporter Activity Is Dispensable for MDA-MB-231 Breast Carcinoma Cell Survival. PloS one. 2014;9(5):e96866. 24802861 10.1371/journal.pone.0096866 PMC4011874

[69] Cain SM, Snutch TP. Voltage‐gated calcium channels and disease. Biofactors. 2011;37(3):197–205. 10.1002/biof.158 21698699

[70] Bidaud I, Mezghrani A, Swayne LA, Monteil A, Lory P. Voltage-gated calcium channels in genetic diseases. Biochimica et Biophysica Acta (BBA)-Molecular Cell Research. 2006;1763(11):1169–74. 17034879 10.1016/j.bbamcr.2006.08.049

[71] Kamp TJ, Hell JW. Regulation of cardiac L-type calcium channels by protein kinase A and protein kinase C. Circulation research. 2000;87(12):1095–102. 11110765 10.1161/01.res.87.12.1095

[72] Moosmang S, Schulla V, Welling A, Feil R, Feil S, Wegener JW, et al Dominant role of smooth muscle L‐type calcium channel Cav1. 2 for blood pressure regulation. The EMBO Journal. 2003;22(22):6027–34. 14609949 10.1093/emboj/cdg583 PMC275441

[73] Skrzypczak M, Goryca K, Rubel T, Paziewska A, Mikula M, Jarosz D, et al Modeling oncogenic signaling in colon tumors by multidirectional analyses of microarray data directed for maximization of analytical reliability. PLoS One. 2010;5(10):e13091 10.1371/journal.pone.0013091 20957034 PMC2948500

[74] Sabates-Bellver J, Van der Flier LG, de Palo M, Cattaneo E, Maake C, Rehrauer H, et al Transcriptome profile of human colorectal adenomas. Molecular cancer research: MCR. 2007;5(12):1263–75. 10.1158/1541-7786.MCR-07-0267 . 18171984

[75] Cho JY, Lim JY, Cheong JH, Park YY, Yoon SL, Kim SM, et al Gene expression signature-based prognostic risk score in gastric cancer. Clinical cancer research: an official journal of the American Association for Cancer Research. 2011;17(7):1850–7. 10.1158/1078-0432.CCR-10-2180 21447720 PMC3078023

[76] D'Errico M, de Rinaldis E, Blasi MF, Viti V, Falchetti M, Calcagnile A, et al Genome-wide expression profile of sporadic gastric cancers with microsatellite instability. European journal of cancer. 2009;45(3):461–9. doi: 10.1016/j.ejca.2008.10.032 . 19081245

[77] Logsdon CD, Simeone DM, Binkley C, Arumugam T, Greenson JK, Giordano TJ, et al Molecular profiling of pancreatic adenocarcinoma and chronic pancreatitis identifies multiple genes differentially regulated in pancreatic cancer. Cancer Res. 2003;63(10):2649–57. . 12750293

[78] Detwiller KY, Fernando NT, Segal NH, Ryeom SW, D'Amore PA, Yoon SS. Analysis of hypoxia-related gene expression in sarcomas and effect of hypoxia on RNA interference of vascular endothelial cell growth factor A. Cancer Res. 2005;65(13):5881–9. 10.1158/0008-5472.CAN-04-4078 . 15994966

[79] Coustan-Smith E, Song G, Clark C, Key L, Liu P, Mehrpooya M, et al New markers for minimal residual disease detection in acute lymphoblastic leukemia. Blood. 2011;117(23):6267–76. 10.1182/blood-2010-12-324004 21487112 PMC3122946

[80] Lee J, Kotliarova S, Kotliarov Y, Li A, Su Q, Donin NM, et al Tumor stem cells derived from glioblastomas cultured in bFGF and EGF more closely mirror the phenotype and genotype of primary tumors than do serum-cultured cell lines. Cancer Cell. 2006;9(5):391–403. 10.1016/j.ccr.2006.03.030 . 16697959

[81] Bredel M, Bredel C, Juric D, Harsh GR, Vogel H, Recht LD, et al Functional network analysis reveals extended gliomagenesis pathway maps and three novel MYC-interacting genes in human gliomas. Cancer Res. 2005;65(19):8679–89. 10.1158/0008-5472.CAN-05-1204 . 16204036

[82] Curtis C, Shah SP, Chin SF, Turashvili G, Rueda OM, Dunning MJ, et al The genomic and transcriptomic architecture of 2,000 breast tumours reveals novel subgroups. Nature. 2012;486(7403):346–52. 10.1038/nature10983 22522925 PMC3440846

[83] Turashvili G, Bouchal J, Baumforth K, Wei W, Dziechciarkova M, Ehrmann J, et al Novel markers for differentiation of lobular and ductal invasive breast carcinomas by laser microdissection and microarray analysis. BMC Cancer. 2007;7:55 10.1186/1471-2407-7-55 17389037 PMC1852112

[84] Quade BJ, Wang TY, Sornberger K, Dal Cin P, Mutter GL, Morton CC. Molecular pathogenesis of uterine smooth muscle tumors from transcriptional profiling. Genes, chromosomes & cancer. 2004;40(2):97–108. 10.1002/gcc.20018 . 15101043

[85] Nindl I, Dang C, Forschner T, Kuban RJ, Meyer T, Sterry W, et al Identification of differentially expressed genes in cutaneous squamous cell carcinoma by microarray expression profiling. Mol Cancer. 2006;5:30 10.1186/1476-4598-5-30 16893473 PMC1569867

[86] LaTulippe E, Satagopan J, Smith A, Scher H, Scardino P, Reuter V, et al Comprehensive gene expression analysis of prostate cancer reveals distinct transcriptional programs associated with metastatic disease. Cancer Res. 2002;62(15):4499–506. . 12154061

[87] Lapointe J, Li C, Higgins JP, van de Rijn M, Bair E, Montgomery K, et al Gene expression profiling identifies clinically relevant subtypes of prostate cancer. Proc Natl Acad Sci U S A. 2004;101(3):811–6. 10.1073/pnas.0304146101 14711987 PMC321763

[88] Taylor BS, Schultz N, Hieronymus H, Gopalan A, Xiao Y, Carver BS, et al Integrative genomic profiling of human prostate cancer. Cancer Cell. 2010;18(1):11–22. 10.1016/j.ccr.2010.05.026 20579941 PMC3198787

[89] Tomlins SA, Mehra R, Rhodes DR, Cao X, Wang L, Dhanasekaran SM, et al Integrative molecular concept modeling of prostate cancer progression. Nat Genet. 2007;39(1):41–51. doi: 10.1038/ng1935 . 17173048

[90] Vanaja DK, Cheville JC, Iturria SJ, Young CY. Transcriptional silencing of zinc finger protein 185 identified by expression profiling is associated with prostate cancer progression. Cancer Res. 2003;63(14):3877–82. . 12873976

[91] Liu P, Ramachandran S, Ali Seyed M, Scharer CD, Laycock N, Dalton WB, et al Sex-determining region Y box 4 is a transforming oncogene in human prostate cancer cells. Cancer Res. 2006;66(8):4011–9. 10.1158/0008-5472.CAN-05-3055 . 16618720

[92] Arredouani MS, Lu B, Bhasin M, Eljanne M, Yue W, Mosquera JM, et al Identification of the transcription factor single-minded homologue 2 as a potential biomarker and immunotherapy target in prostate cancer. Clinical cancer research: an official journal of the American Association for Cancer Research. 2009;15(18):5794–802. 10.1158/1078-0432.CCR-09-0911 . 19737960 PMC5573151

[93] Wallace TA, Prueitt RL, Yi M, Howe TM, Gillespie JW, Yfantis HG, et al Tumor immunobiological differences in prostate cancer between African-American and European-American men. Cancer Res. 2008;68(3):927–36. 10.1158/0008-5472.CAN-07-2608 . 18245496

[94] Luo JH, Yu YP, Cieply K, Lin F, Deflavia P, Dhir R, et al Gene expression analysis of prostate cancers. Mol Carcinogen. 2002;33(1):25–35. 10.1002/Mc.10018 . 11807955

[95] Magee JA, Araki T, Patil S, Ehrig T, True L, Humphrey PA, et al Expression profiling reveals hepsin overexpression in prostate cancer. Cancer Research. 2001;61(15):5692–6. . 11479199

[96] Radvanyi L, Singh-Sandhu D, Gallichan S, Lovitt C, Pedyczak A, Mallo G, et al The gene associated with trichorhinophalangeal syndrome in humans is overexpressed in breast cancer. Proceedings of the National Academy of Sciences of the United States of America. 2005;102(31):11005–10. 10.1073/pnas.0500904102 . 16043716 PMC1182410

[97] Dyrskjot L, Kruhoffer M, Thykjaer T, Marcussen N, Jensen JL, Moller K, et al Gene expression in the urinary bladder: a common carcinoma in situ gene expression signature exists disregarding histopathological classification. Cancer Res. 2004;64(11):4040–8. 10.1158/0008-5472.CAN-03-3620 . 15173019

[98] Cui JA, Chen YB, Chou WC, Sun LK, Chen L, Suo JA, et al An integrated transcriptomic and computational analysis for biomarker identification in gastric cancer. Nucleic Acids Res. 2011;39(4):1197–207. 10.1093/Nar/Gkq960 . 20965966 PMC3045610

[99] Bhattacharjee A, Richards WG, Staunton J, Li C, Monti S, Vasa P, et al Classification of human lung carcinomas by mRNA expression profiling reveals distinct adenocarcinoma subclasses. Proc Natl Acad Sci U S A. 2001;98(24):13790–5. 10.1073/pnas.191502998 11707567 PMC61120

[100] Bhat S, Dao DT, Terrillion CE, Arad M, Smith RJ, Soldatov NM, et al CACNA1C (Ca(v)1.2) in the pathophysiology of psychiatric disease. Prog Neurobiol. 2012;99(1):1–14. 10.1016/j.pneurobio.2012.06.001 . 22705413 PMC3459072

[101] Chambers KF, Pearson JF, Pellacani D, Aziz N, Gužvić M, Klein CA, et al Stromal upregulation of lateral epithelial adhesions: Gene expression analysis of signalling pathways in prostate epithelium. Journal of biomedical science. 2011;18(1):1–13. 21696611 10.1186/1423-0127-18-45 PMC3141633

[102] Szatkowski C, Parys JB, Ouadid-Ahidouch H, Matifat F. Inositol 1, 4, 5-trisphosphate-induced Ca2+ signalling is involved in estradiol-induced breast cancer epithelial cell growth. Molecular Cancer. 2010;9(1):156. 20565939 10.1186/1476-4598-9-156 PMC2906470

[103] Pesch K, Zeitz C, Fries JE, Münscher S, Pusch CM, Kohler K, et al Isolation of the mouse nyctalopin gene nyx and expression studies in mouse and rat retina. Investigative ophthalmology & visual science. 2003;44(5):2260–6. 12714669 10.1167/iovs.02-0115

[104] Llinas R, Sugimori M, Lin J, Cherksey B. Blocking and isolation of a calcium channel from neurons in mammals and cephalopods utilizing a toxin fraction (FTX) from funnel-web spider poison. Proceedings of the National Academy of Sciences. 1989;86(5):1689–93. 10.1073/pnas.86.5.1689 PMC286766 2537980

[105] Randall A, Tsien RW. Pharmacological dissection of multiple types of Ca2+ channel currents in rat cerebellar granule neurons. The Journal of neuroscience. 1995;15(4):2995–3012. 7722641 10.1523/JNEUROSCI.15-04-02995.1995 PMC6577783

[106] Bourinet E, Soong TW, Sutton K, Slaymaker S, Mathews E, Monteil A, et al Splicing of α1A subunit gene generates phenotypic variants of P-and Q-type calcium channels. Nature neuroscience. 1999;2(5):407–15. 10321243 10.1038/8070

[107] Chaudhuri D, Chang S-Y, DeMaria CD, Alvania RS, Soong TW, Yue DT. Alternative splicing as a molecular switch for Ca2+/calmodulin-dependent facilitation of P/Q-type Ca2+ channels. The Journal of neuroscience. 2004;24(28):6334–42. 15254089 10.1523/JNEUROSCI.1712-04.2004 PMC6729554

[108] Gazulla J, Tintore M. P/Q-type voltage-dependent calcium channels in neurological disease. Neurologia. 2007;22(8):511–6. . 17573560

[109] Guida S, Trettel F, Pagnutti S, Mantuano E, Tottene A, Veneziano L, et al Complete loss of P/Q calcium channel activity caused by a CACNA1A missense mutation carried by patients with episodic ataxia type 2. The American Journal of Human Genetics. 2001;68(3):759–64. 11179022 10.1086/318804 PMC1274487

[110] Battistini S, Stenirri S, Piatti M, Gelfi C, Righetti P, Rocchi R, et al A new CACNA1A gene mutation in acetazolamide-responsive familial hemiplegic migraine and ataxia. Neurology. 1999;53(1):38–. 10408534 10.1212/wnl.53.1.38

[111] Segarra NG, Gautschi I, Mittaz-Crettol L, Zetchi CK, Al-Qusairi L, Van Bemmelen MX, et al Congenital ataxia and hemiplegic migraine with cerebral edema associated with a novel gain of function mutation in the calcium channel CACNA1A. Journal of the neurological sciences. 2014;342(1):69–78. 24836863 10.1016/j.jns.2014.04.027

[112] Kono S, Terada T, Ouchi Y, Miyajima H. An altered GABA-A receptor function in spinocerebellar ataxia type 6 and familial hemiplegic migraine type 1 associated with the CACNA1A gene mutation. BBA Clinical. 2014;2:56–61. 26675662 10.1016/j.bbacli.2014.09.005 PMC4633947

[113] Tothill RW, Tinker AV, George J, Brown R, Fox SB, Lade S, et al Novel molecular subtypes of serous and endometrioid ovarian cancer linked to clinical outcome. Clinical Cancer Research. 2008;14(16):5198–208. 10.1158/1078-0432.Ccr-08-0196 . 18698038

[114] Haferlach T, Kohlmann A, Wieczorek L, Basso G, Kronnie GT, Bene MC, et al Clinical Utility of Microarray-Based Gene Expression Profiling in the Diagnosis and Subclassification of Leukemia: Report From the International Microarray Innovations in Leukemia Study Group. J Clin Oncol. 2010;28(15):2529–37. 10.1200/Jco.2009.23.4732 . 20406941 PMC5569671

[115] Zhan FH, Barlogie B, Arzoumanian V, Huang YS, Hollmig K, Pineda-Roman M, et al A gene expression signature of benign monoclonal gammopathy evident in multiple myeloma is linked to good prognosis. Blood. 2006;108(11):969a–a. . 10.1182/blood-2006-07-037077 PMC1794073 17023574

[116] Riker AI, Enkemann SA, Fodstad O, Liu S, Ren S, Morris C, et al The gene expression profiles of primary and metastatic melanoma yields a transition point of tumor progression and metastasis. BMC medical genomics. 2008;1(1):13. 18442402 10.1186/1755-8794-1-13 PMC2408576

[117] Zhai Y, Kuick R, Nan B, Ota I, Weiss SJ, Trimble CL, et al Gene expression analysis of Preinvasive and invasive cervical squamous cell carcinomas identifies HOXC10 as a key mediator of invasion. Cancer Research. 2007;67(21):10163–72. 10.1158/0008-5472.Can-07-2056 . 17974957

[118] Su AI, Welsh JB, Sapinoso LM, Kern SG, Dimitrov P, Lapp H, et al Molecular classification of human carcinomas by use of gene expression signatures. Cancer research. 2001;61(20):7388–93. 11606367

[119] Su L-J, Chang C-W, Wu Y-C, Chen K-C, Lin C-J, Liang S-C, et al Selection of DDX5 as a novel internal control for Q-RT-PCR from microarray data using a block bootstrap re-sampling scheme. BMC genomics. 2007;8(1):140. 17540040 10.1186/1471-2164-8-140 PMC1894975

[120] Varambally S, Yu JJ, Laxman B, Rhodes DR, Mehra R, Tomlins SA, et al Integrative genomic and proteomic analysis of prostate cancer reveals signatures of metastatic progression. Cancer Cell. 2005;8(5):393–406. 10.1016/j.ccr.2005.10.001 . 16286247

[121] Lory P, Bidaud I, Chemin J. T-type calcium channels in differentiation and proliferation. Cell calcium. 2006;40(2):135–46. 16797068 10.1016/j.ceca.2006.04.017

[122] Bertolesi GE, Shi C, Elbaum L, Jollimore C, Rozenberg G, Barnes S, et al The Ca2+ channel antagonists mibefradil and pimozide inhibit cell growth via different cytotoxic mechanisms. Molecular pharmacology. 2002;62(2):210–9. 12130671 10.1124/mol.62.2.210

[123] Chambers AF, Groom AC, MacDonald IC. Metastasis: dissemination and growth of cancer cells in metastatic sites. Nature Reviews Cancer. 2002;2(8):563–72. 12154349 10.1038/nrc865

[124] Hahnfeldt P, Panigrahy D, Folkman J, Hlatky L. Tumor development under angiogenic signaling a dynamical theory of tumor growth, treatment response, and postvascular dormancy. Cancer research. 1999;59(19):4770–5. 10519381

[125] Allard WJ, Matera J, Miller MC, Repollet M, Connelly MC, Rao C, et al Tumor cells circulate in the peripheral blood of all major carcinomas but not in healthy subjects or patients with nonmalignant diseases. Clinical Cancer Research. 2004;10(20):6897–904. 15501967 10.1158/1078-0432.CCR-04-0378

[126] Padera TP, Kadambi A, di Tomaso E, Carreira CM, Brown EB, Boucher Y, et al Lymphatic metastasis in the absence of functional intratumor lymphatics. Science. 2002;296(5574):1883–6. 11976409 10.1126/science.1071420

[127] Friedl P, Gilmour D. Collective cell migration in morphogenesis, regeneration and cancer. Nature reviews Molecular cell biology. 2009;10(7):445–57. 10.1038/nrm2720 19546857

[128] Weigelt B, Peterse JL, Van't Veer LJ. Breast cancer metastasis: markers and models. Nature reviews cancer. 2005;5(8):591–602. 16056258 10.1038/nrc1670

[129] Winston CB, Hadar O, Teitcher JB, Caravelli JF, Sklarin NT, Panicek DM, et al Metastatic lobular carcinoma of the breast: patterns of spread in the chest, abdomen, and pelvis on CT. American Journal of Roentgenology. 2000;175(3):795–800. 10954469 10.2214/ajr.175.3.1750795

[130] Borst M, Ingold J. Metastatic patterns of invasive lobular versus invasive ductal carcinoma of the breast. Surgery. 1993;114(4):637–41; discussion 41–2. 8211676

[131] Harris M, Howell A, Chrissohou M, Swindell R, Hudson M, Sellwood R. A comparison of the metastatic pattern of infiltrating lobular carcinoma and infiltrating duct carcinoma of the breast. British journal of cancer. 1984;50(1):23 6331484 10.1038/bjc.1984.135 PMC1976917

[132] Luo W, Chang R, Zhong J, Pandey A, Semenza GL. Histone demethylase JMJD2C is a coactivator for hypoxia-inducible factor 1 that is required for breast cancer progression. Proceedings of the National Academy of Sciences. 2012;109(49):E3367–E76. 10.1073/pnas.1217394109 PMC3523832 23129632

[133] Shinagare AB, Ramaiya NH, Jagannathan JP, Fennessy FM, Taplin M-E, Van den Abbeele AD. Metastatic pattern of bladder cancer: correlation with the characteristics of the primary tumor. American Journal of Roentgenology. 2011;196(1):117–22. 10.2214/AJR.10.5036 21178055

[134] Schuchert MJ, Luketich JD. Solitary sites of metastatic disease in non-small cell lung cancer. Current treatment options in oncology. 2003;4(1):65–79. 12525281 10.1007/s11864-003-0033-8

[135] Coghlin C, Murray GI. Current and emerging concepts in tumour metastasis. The Journal of pathology. 2010;222(1):1–15. 10.1002/path.2727 20681009

[136] Aragon-Ching JB, Zujewski JA. CNS metastasis: an old problem in a new guise. Clinical Cancer Research. 2007;13(6):1644–7. 17363516 10.1158/1078-0432.CCR-07-0096

[137] Javier BV, Yount WJ, Crosby DJ, Hall TC. Cardiac metastasis in lymphoma and leukemia. CHEST Journal. 1967;52(4):481–4. 10.1378/chest.52.4.481 4862352

[138] Krenek P, Kmecova J, Kucerova D, Bajuszova Z, Musil P, Gazova A, et al Isoproterenol-induced heart failure in the rat is associated with nitric oxide-dependent functional alterations of cardiac function. European journal of heart failure. 2009;11(2):140–6. 10.1093/eurjhf/hfn026 19168511 PMC2639419

[139] Chen R, Zeng X, Zhang R, Huang J, Kuang X, Yang J, et al Cav1.3 channel α1D protein is overexpressed and modulates androgen receptor transactivation in prostate cancers. Urologic Oncology: Seminars and Original Investigations. 2014;32(5):524–36. doi: 10.1016/j.urolonc.2013.05.011. 10.1016/j.urolonc.2013.05.011 24054868

[140] Choi DL, Jang SJ, Cho S, Choi H-E, Rim H-K, Lee K-T, et al Inhibition of cellular proliferation and induction of apoptosis in human lung adenocarcinoma A549 cells by T-type calcium channel antagonist. Bioorganic & medicinal chemistry letters. 2014;24(6):1565–70. 24529871 10.1016/j.bmcl.2014.01.071

[141] Chen Q, Zhang Q, Zhong F, Guo S, Jin Z, Shi W, et al Association between calcium channel blockers and breast cancer: a meta-analysis of observational studies. Pharmacoepidemiology and Drug Safety. 2014;23(7):711–8. 10.1002/pds.3645 24829113

[142] Pierro C, Cook SJ, Foets TC, Bootman MD, Roderick HL. Oncogenic K-Ras suppresses IP3-dependent Ca2+ release through remodelling of the isoform composition of IP3Rs and ER luminal Ca2+ levels in colorectal cancer cell lines. Journal of cell science. 2014;127(7):1607–19. 24522186 10.1242/jcs.141408

