# Supplementary figures and images for: Meta-Analysis of Public Microarray Datasets Reveals Voltage-Gated Calcium Gene Signatures in Clinical Cancer Patients
Source: PLoS One. 2015 Jul 6;10(7):e0125766. doi: 10.1371/journal.pone.0125766 (PMC4493072; doi:10.1371/journal.pone.0125766)

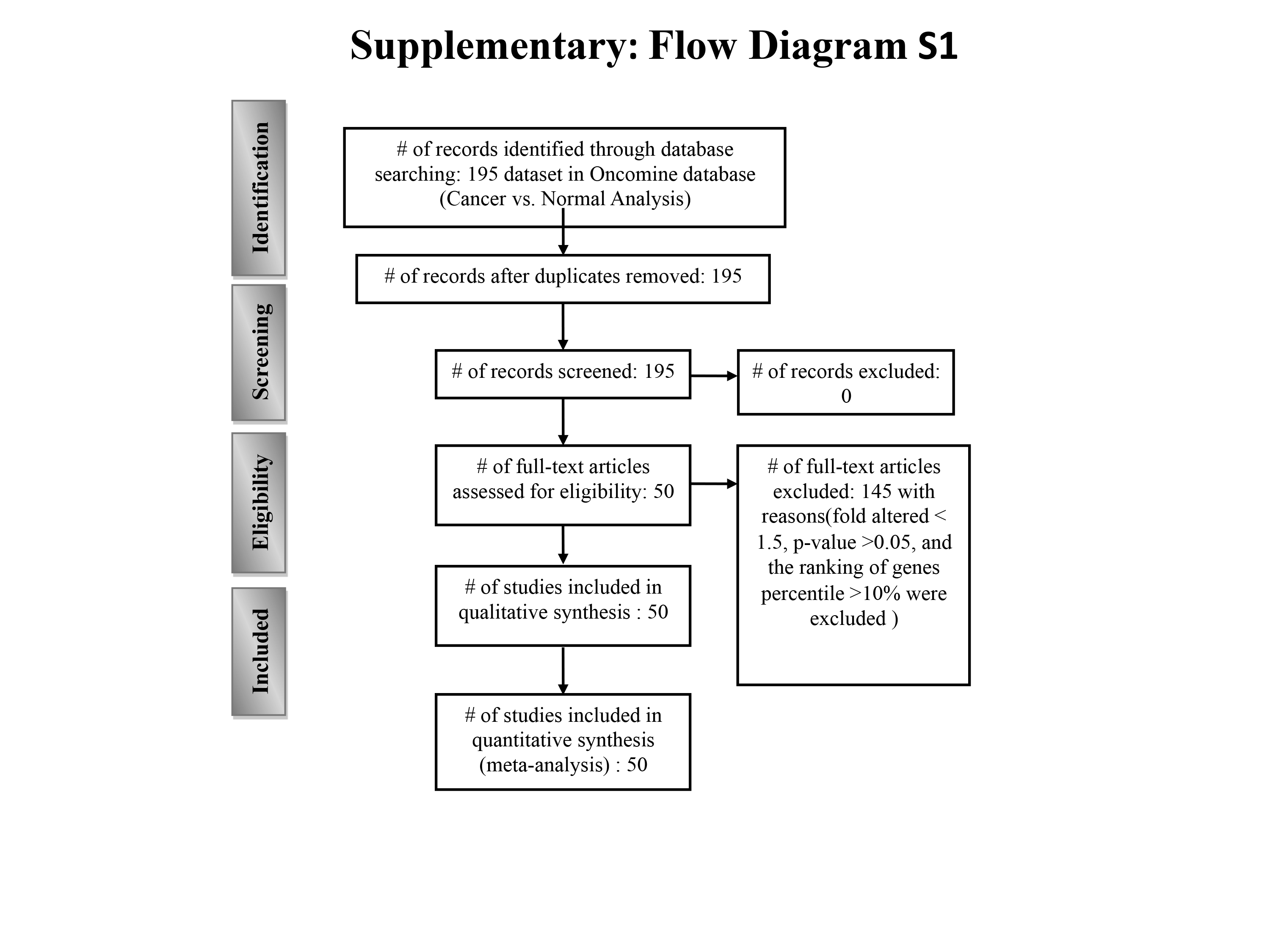

Supplement: S1 Fig — (TIF) [file pone.0125766.s001.tif]

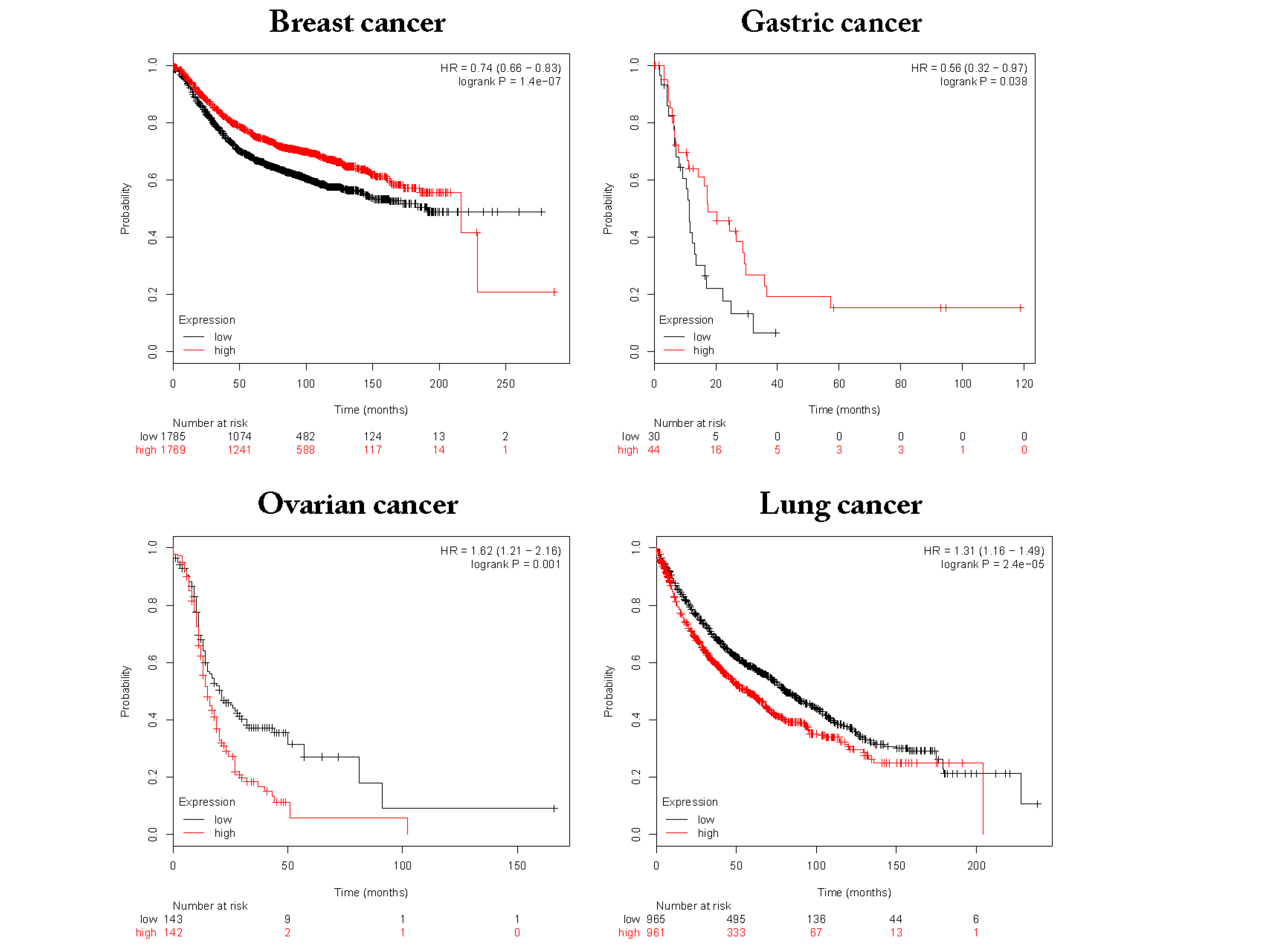

Supplement: S2 Fig — Kaplan-Meier plots showing overall survival in breast, gastric, ovarian and lung cancer. Over-expression of CACNA1A in ovarian and lung cancer would cause poor prognosis, whereas in breast and gastric it would lead to good prognosis. Breast cancer, p = 1.4X 10–7; gastric cancer, p = 0.038; ovarian cancer, p = 0.001; lung cancer, p = 2.4 X10-5. (TIF) [file pone.0125766.s002.tif]

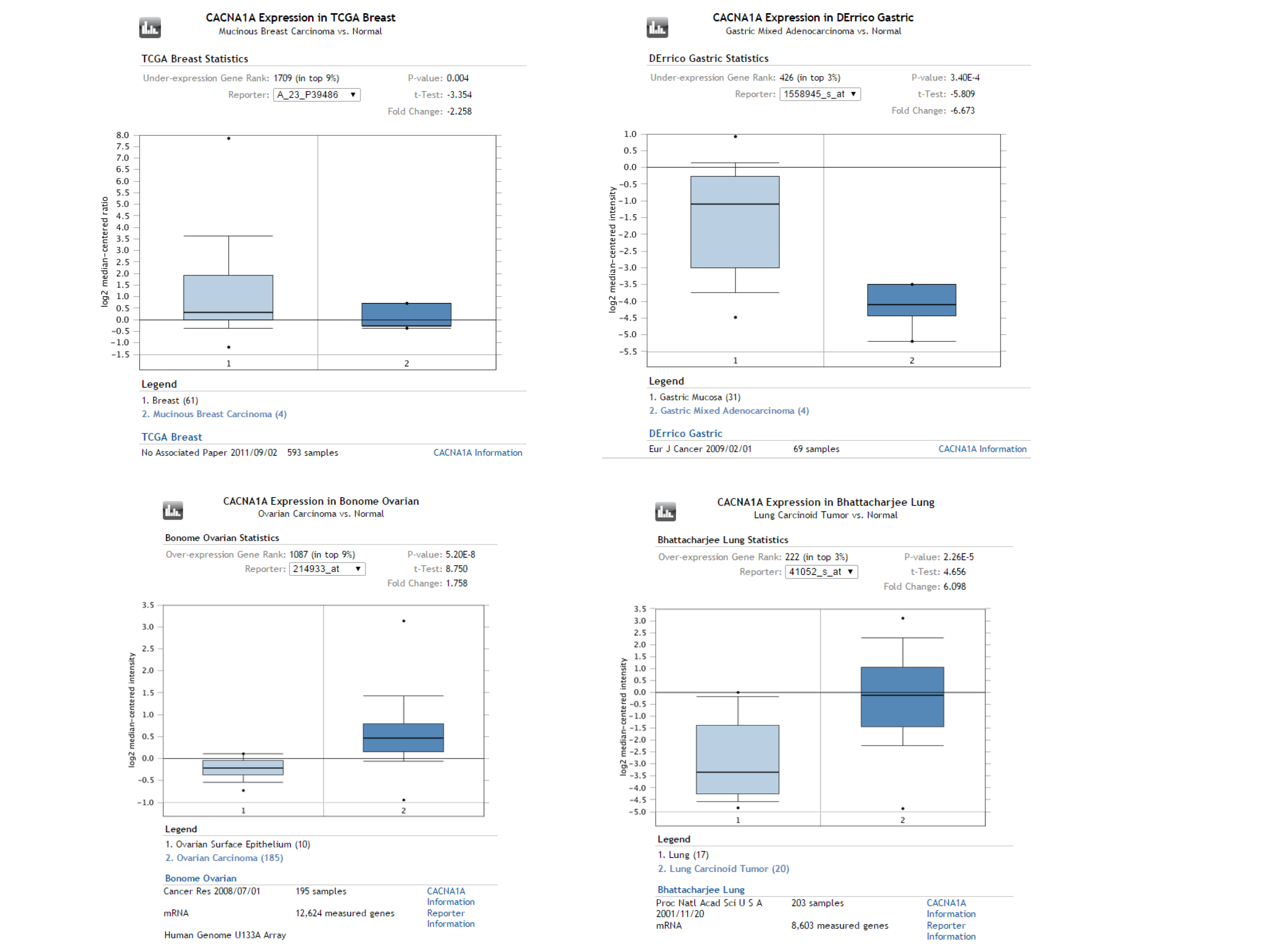

Supplement: S3 Fig — Box plots derived from gene expression data in ONCOMINE comparing expression of the CACNA1A gene in normal (left plot) and various types of cancer tissue (right plot). (TIF) [file pone.0125766.s003.tif]
