# Supplementary material for: Meta-Analysis of Public Microarray Datasets Reveals Voltage-Gated Calcium Gene Signatures in Clinical Cancer Patients
Source: PLoS One. 2015 Jul 6;10(7):e0125766. doi: 10.1371/journal.pone.0125766 (PMC4493072; doi:10.1371/journal.pone.0125766)
Supplement: S2 Table — (DOCX) [file pone.0125766.s005.docx]

| Ref | Journal and date | Oncomine dataset | Study Description |
| --- | --- | --- | --- |
| [[1](#_ENREF_1)] | Nature 2002/01/24 | Pomeroy Brain | Eighty-one (81) brain and CNS cancer and 4 normal cerebellum samples were analyzed on Affymetrix HuGeneFL microarrays. Sample data includes type, age, histologic subtype, M stage, metastasis, sex, survival, and T stage. |
| [[2](#_ENREF_2)] | Nat Genet 2004/03/01 | Stegmaier Leukemia | Eighty-one (81) leukemia (60 of which were the acute promyelocytic leukemia cell line HL-60 and 12 of which were acute promyelocytic leukemia cell cultures) and 6 normal blood samples were analyzed on Affymetrix microarrays. Sample data includes type and treatment. |
| [[3](#_ENREF_3)] | PLoS One 2010/10/01 | Skrzypczak Colorectal | Thirty-six macro-dissected (36) colorectal carcinoma, 45 colorectal adenocarcinoma, and 24 normal colorectal tissues were analyzed. Corresponding micro-dissected data from the same publication is available in Skrzypczak Colorectal 2. |
| [[4](#_ENREF_4)] | Mol Cancer Res 2007/12/01 | Sabates-Bellver Colon | Early, sporadic, pedunculated colorectal carcinomas of Type 0-1p (Paris Classification) (n=32) and matched normal colon samples (n=32) were analyzed on Affymetrix Human Genome U133 Plus 2.0 microarrays. Sample data includes tumor location, tumor size, and patient ID. |
| [[5](#_ENREF_5)] | Clin Cancer Res 2011/04/01 | Cho Gastric | Sixty-five (65) gastric adenocarcinoma, 19 paired surrounding normal tissue, and 6 gastrointestinal stromal tumor samples were analyzed. Sample data includes age, grade, stage, TNM stage, sex, and subgroup. |
| [[6](#_ENREF_6)] | Cancer Res 2003/05/15 | Logsdon Pancreas | Seventeen (17) pancreatic carcinoma (including 7 cell lines), 5 pancreatitis, and 5 normal pancreas samples were analyzed on Affymetrix HuGeneFL microarrays. Sample data includes type and cell line name. |
| [[7](#_ENREF_7)] | Cancer Res 2005/07/01 | Detwiller Sarcoma | Thirty-nine (39) sarcoma and 15 normal tissue samples of various types were analyzed on Affymetrix U133A microarrays. |
| [[8](#_ENREF_8)] | Blood 2011/06/09 | Coustan-Smith Leukemia S | Two hundred thirty-eight (238) B-cell childhood acute lymphoblastic leukemia, 46 T-cell childhood acute lymphoblastic leukemia, and 4 CD10-positive CD19-positive hematogone samples were analyzed. Sample data includes gene fusion status for BCR-ABL1, ETV6-RUNX1, TCF3-PBX1, MLL rearrangement status, and ploidy status. |
| [[9](#_ENREF_9)] | Cancer Cell 2006/05/01 | Lee Brain | Ninety-eight (98) brain and CNS cancer (26 of which are cell lines) and 3 normal neural stem cell samples were analyzed on Affymetrix U133 Plus 2.0 microarrays. Sample data includes type, growth media, cell line name, and passage. |
| [[10](#_ENREF_10)] | Cancer Res 2005/10/01 | Bredel Brain | Fifty (50) brain CNS carcinoma samples and 4 normal brain samples were analyzed on cDNA microarrays. Sample data includes disease type. |
| [[11](#_ENREF_11)] | Nature 2012/04/18 | Curtis Breast | One thousand nine hundred nintey-two (1,992) breast carcinoma samples and 144 paired normal breast samples were analyzed for the METABRIC project. Sample data includes ER/PR/ERBB2 status, overall survival status and followup time, stage, grade, and others. Corresponding DNA copy number data is available in Curtis Breast 2. |
| [[12](#_ENREF_12)] | BMC Cancer 2007/03/27 | Turashvili Breast | Tissue samples from breast carcinomas of the duct (n=5) and lobules (n=5), as well as normal adjacent duct (n=10) and normal adjacent lobules (n=10) were analyzed on Affymetrix HG U133 Plus 2.0 arrays. Sample data includes cancer type, Nottingham grade, accompanying changes, lymph node status, estrogen receptor status, progesterone receptor status, HER2 status, Bcl-2 status, patient ID, and E-cadherin status. |
| [[13](#_ENREF_13)] | Genes Chromosomes Cancer 2004/06/01 | Quade Uterus | Twenty (20) smooth muscle neoplasms and 4 normal myometrium samples were analyzed on Affymetrix HuGeneFL microarrays. Sample data includes type, age, karyotype, and patient ID. |
| [[14](#_ENREF_14)] | Mol Cancer 2006/08/08 | Nindl Skin | Normal skin, actinic keratinocytes, and invasive squamous cell carcinoma samples were collected from 5 immunosuppressed organ-transplant recipients; for microarray analysis, the 5 normal samples were pooled, as were two of the actinic keratinocytes samples. In addition, 5 normal skin samples from immunocompetent individuals were collected and analyzed. Altogether, fifteen (15) total samples were analyzed (normal skin (n=6), actinic keratosis (n=4), and squamous cell carcinoma (n=5) on Affymetrix U133A microarrays. |
| [[15](#_ENREF_15)] | Cancer Res 2002/08/01 | LaTulippe Prostate | Thirty-two (32) prostate carcinoma and 3 normal prostate gland samples were analyzed on Affymetrix U95A microarrays. Sample data includes type, age, Gleason score, TNM stage, PSA recurrence, previous treatment, race, site, and site of metastasis. |
| [[16](#_ENREF_16)] | Proc Natl Acad Sci U S A 2004/01/20 | Lapointe Prostate | Sixty-two (62) prostate carcinomas, forty-one (41) matched normal prostate, and nine (9) metastatic prostate cancer in lymph node samples were analyzed on cDNA microarrays. Sample data includes tumor type, age, grade, stage, recurrence and survival. |
|  | [TCGA Breast](https://www.oncomine.org/resource/ui/component/dataset.html?component=d:156636492) |  | Five hundred thirty-two (532) invasive breast carcinoma, 61 paired normal breast tissue and 3 paired metastatic samples were analyzed. Sample data includes age, histology, TNM stage, ER/PR/ERBB2 status, sex, stage, and others. This dataset consists of Level 2 (processed) data from the TCGA data portal. Corresponding DNA copy number data is available in TCGA Breast 2. |
| [[17](#_ENREF_17)] | Nature 2012/05/20 | Grasso Prostate | Thirty-five (35) castrate-resistant metastatic prostate cancer, 59 localized prostate carcinoma, and 28 benign prostate tissue specimens were analyzed. Sample data includes overall survival, abberations in the ETS family, PTEN deletion, AR amplification, select mutations from the exome sequencing data, and others. Corresponding DNA copy number data is available in Grasso Prostate 2. |
| [[18](#_ENREF_18)] | Cancer Cell 2010/07/13 | Taylor Prostate | One hundred fifty (150) prostate carcinoma tissue specimens (including 131 specimens from primary tumors and 19 metastases), 29 paired normal adjacent prostate tissue specimens, and 6 cell lines were analyzed. Sample data includes T stage, N stage, race, age, Gleason score, biochemical recurrence, and overall survival status. Corresponding DNA copy number data available in Taylor Prostate. |
| [[19](#_ENREF_19)] | Nat Genet 2007/01/01 | Tomlins Prostate | 101 laser dissected cell populations from a progression of prostate cancer cell types (benign, PIN, low-grade PCA, high-grade PCA, metastatic PCA) and normal adjacent tissue were analyzed with a custom microarray chip. |
| [[3](#_ENREF_3)] | PLoS One 2010/10/01 | Skrzypczak Colorectal 2 | Forty (40) microdissected samples (5 replicates each of 8 types of epithelial or mucosa cells from tumor or normal tissues) were analyzed. Corresponding macro-dissected data from the same publication is available in Skrzypczak Colorectal. |
| [[20](#_ENREF_20)] | Cancer Res 2003/07/15 | Vanaja Prostate | Thirty-two (32) prostate adenocarcinoma and 8 normal prostate gland samples were analyzed on Affymetrix U133A/B microarrays. Sample data includes type, percent tumor, age, grade, and T stage. |
| [[21](#_ENREF_21)] | Cancer Res 2004/06/01 | Dyrskjot Bladder 3 | Sixty (60) bladder samples, including 14 normal bladder, 5 CIS, 28 superficial bladder cancer, and 13 invasive bladder cancer samples, were analyzed on Affymetrix U133A microarrays. Sample data includes type, grade, T stage, and CIS details. |
| [[22](#_ENREF_22)] | Cancer Res 2006/04/15 | Liu Prostate | Fifty-seven (57) prostate samples, including 44 prostate carcinoma and 13 adjacent normal samples, were analyzed on Affymetrix U133A microarrays. Sample data includes Gleason score, T stage, patient ID, and age. |
|  | TCGA Colorectal |  | Two hundred fifteen (215) colorectal adenocarcinoma and 22 paired normal colorectal tissue samples were analyzed. Sample data includes age, histology, microsatellite status, TNM stage, KRAS mutation status, sex, stage, and others. This dataset is a combination of Colon Adenocarcinoma [COAD] and Rectum Adenocarcinoma [READ] data from the TCGA data portal and consists of Level 2 (processed) data. Corresponding DNA copy number data is available in TCGA Colorectal 2. |
| [[23](#_ENREF_23)] | Clin Cancer Res 2009/09/15 | Arredouani Prostate | Thirteen (13) prostate carcinoma samples and 8 normal prostate were analyzed. Sample data includes ERG rearrangement. |
| [[24](#_ENREF_24)] | Eur J Cancer 2009/02/01 | DErrico Gastric | Thirty-one (31) paired gastric carcinoma and adjacent normal gastric mucosa and 7 unmatched gastric carcinoma samples were analyzed. Sample data includes microsatellite status, age, sex, and TNM stage. |
| [[25](#_ENREF_25)] | Proc Natl Acad Sci U S A 2001/11/20 | Bhattacharjee Lung | One hundred thirty-nine (139) lung adenocarcinoma, 21 squamous cell lung carcinoma, 20 lung carcinoid tumor, 6 small cell lung carcinoma, and 17 normal lung samples were analyzed on Affymetrix U95A microarrays. Sample data includes type, age, M stage, max tumor percentage, N stage, primary/metastatic, recurrence, sex, site of metastasis, smoking rate (packs per year), stage, survival, and T stage. |
| [[26](#_ENREF_26)] | Cancer Res 2008/02/01 | Wallace Prostate | Sixty-nine (69) prostate tumor samples, 18 adjacent normal prostate samples, and 2 pooled normal prostate samples were analyzed on Affymetrix HG-U133A 2.0 microarrays. Sample data includes race, smoking status, Gleason score, pathological T stage, extraprostatic extension, perineural invasion, seminal vesicle invasion, surgical margin involvement, patient ID, and angio-lymphatic invasion. |
| [[27](#_ENREF_27)] | Cancer Res 2009/08/01 | Crabtree Uterus | Matched uterine corpus leiomyoma and normal myometrium samples from 23 patients were analyzed. Many samples were run in duplicate or triplicate. |
| [[28](#_ENREF_28)] | Gastroenterology 2006/09/01 | Hao Esophagus | Forty-eight (48) samples from 17 patients were analyzed on cDNA microarrays. Tissue types include normal esophagus (n=15), normal duodenum (n=14), Barretts esophagus (n=14), and esophageal adenocarcinoma (n=5). Sample data includes age, race, sex, patient ID, and Barretts esophagus length. |
| [[29](#_ENREF_29)] | Nucleic Acids Res 2011/03/01 | Cui Gastric | Eighty (80) gastric carcinoma and 80 normal paired gastric tissue samples were analyzed. Sample data includes age, sex, stage, and grade. |
| [[30](#_ENREF_30)] | Mol Carcinog 2002/01/01 | Luo Prostate 2 | Fifteen (15) prostate carcinoma and 15 matched normal prostate gland samples were analyzed on Affymetrix microarrays. Sample data includes type, Gleason score, patient ID, and T stage. |
| [[31](#_ENREF_31)] | Proc Natl Acad Sci U S A 2005/08/02 | Radvanyi Breast | Fifty-four (54) breast carcinoma and 9 normal breast samples were analyzed on Protein Design Labs Hu03 Custom Affymetrix GeneChip microarrays. Sample data includes type, age, grade, lymph node involvement, nuclear pleiomorphisms, sex, surgical margins, tissue, tubule formation, and tumor size. |
| [[32](#_ENREF_32)] | Cancer Res 2001/08/01 | Magee Prostate | Eleven (11) prostate carcinoma (8 primary and 3 from metastasis) and 4 normal prostate gland samples were analyzed on Affymetrix Hu6800 microarrays. |
| [[33](#_ENREF_33)] | Cancer Res 2010/12/15 | Roessler Liver | Two hundred twenty-five (225) hepatocellular carcinoma and 220 normal liver samples were analyzed. Additional data from this paper can be found in Roessler Liver. |
| [[34](#_ENREF_34)] | Cancer Res 2005/07/01 | Skotheim Testis | A total of 29 tissue samples were analyzed on Agilent 60mer oligo microarrays. Twenty-three (23) of the samples are from testicular germ cell tumors (premalignant IGCN (n=3), seminoma (n=3), embryonal carcinoma (n=5), yolk sac tumor (n=4), choriocarcinoma (n=1), teratoma (n=4), and normal testicular parenchyma (n=3)). Additional data is provided on 6 samples from 2 embryonal carcinoma cell lines, each treated for 0, 3, or 7 days with Retinoic Acid. |
| [[35](#_ENREF_35)] | J Clin Oncol 2010/05/20 | Haferlach Leukemia | Purified mononuclear cell samples from two thousand ninety-six (2,096) leukemia samples were analyzed as part of the Microarray Innovations in Leukemia study program. Study authors define seventeen leukemia and one normal/non-leukemia subtype that are available in Oncomine for Group By or data export. The normal/non-leukemia subtype is made up of healthy bone marrow specimens or a variety of non-leukemia conditions (e.g. hemolysis). Sample data includes diagnosis, karyotype, and translocation/fusion status. An additional 1,152 samples run on a custom chip are available in Haferlach Leukemia 2. |
| [[36](#_ENREF_36)] | Cancer Res 2008/07/01 | Bonome Ovarian | One hundred eighty-five (185) ovarian carcinoma samples and 10 normal ovarian surface epithelium samples were analyzed. Sample metadata includes overall survival, grade, and stage. |
| [[37](#_ENREF_37)] | Nat Genet 2010/07/04 | Barretina Sarcoma | One hundred forty-nine (149) soft tissue sarcomas of 6 different subtypes and 9 normal adipose tissue specimens were analyzed. Sample data includes karyotype and mutation status for CDH1, CTNNB1, EPHA1, EPHA5, EPHA7, ERBB4, FBXW7, IRS1, KIT, LTK, MOS, MST1R, NF1, NTRK1, PI4KA, PIK3CA, PTEN, PTK2B, RB1, SYK, and TP53. Corresponding DNA copy number data is available in Barretina Sarcoma 2. |
| [[38](#_ENREF_38)] | Blood 2007/02/15 | Zhan Myeloma 3 | Twenty-two (22) normal bone marrow, forty-four (44) monoclonal gammopathy of undetermined significance, and twelve (12) multiple myeloma samples were analyzed on Affymetrix U133 Plus 2.0 microarrays. |
|  | [TCGA Ovarian](https://www.oncomine.org/resource/ui/component/dataset.html?component=d:111287677) | TCGA Ovarian | Five hundred eighty-six (586) ovarian serous cystadenocarcinoma samples and 8 normal ovary samples were analyzed. Sample data includes age, stage, grade, survival, and others. This dataset consists of Level 2 (processed) data from the TCGA data portal. Corresponding DNA copy number data is available in TCGA Ovarian 2. |
| [[39](#_ENREF_39)] | BMC Med Genomics 2008/04/28 | Riker Melanoma | Forty (40) metastatic melanoma, 42 primary skin cancer, 4 normal skin, and 1 normal skin primary cell culture samples were analyzed on Affymetrix HG U133 Plus 2.0 microarrays. Sample data includes type and Breslow thickness(mm). |
| [[40](#_ENREF_40)] | J Invest Dermatol 2003/05/01 | Storz Lymphoma | Nineteen (19) lymphoma, 2 mycosis fungoides, 3 normal skin, and 3 normal tonsil samples were analyzed on cDNA microarrays. Sample data includes type, age, biopsy site, and sex. |
| [[41](#_ENREF_41)] | Cancer Res 2007/11/01 | Zhai Cervix | Twenty-one (21) cervical squamous cell carcinoma, 7 high grade cervical squamous intraepithelial neoplasia and 10 normal cervical squamous epithelia samples were analyzed. Sample data includes human papillomavirus infection status. |
| [[42](#_ENREF_42)] | BMC Genomics 2007/06/01 | Su Lung | Sixty-six (66) lung samples were analyzed on Affymetrix U133A microarrays. Samples include twenty-six lung adenocarcinomas with paired adjacent normals, one large cell lung carcinoma with paired adjacent normal, two tissue mixtures, two commercial human normal lung tissues, one normal lung cell line, and seven lung cancer cell lines. |
| [[43](#_ENREF_43)] | Cancer Cell 2005/11/01 | Varambally Prostate | Six (6) hormone-refractory metastatic prostate carcinoma samples, 7 primary prostate carcinoma samples, and 6 normal prostate gland samples were analyzed. |
| [[44](#_ENREF_44)] | Clin Cancer Res 2005/11/15 | Cutcliffe Renal | Eighteen (18) Wilms tumor samples, 14 clear cell sarcoma of the kidney samples, and 3 normal fetal kidney samples were analyzed on Affymetrix U133A microarrays. |
| [[45](#_ENREF_45)] | Nat Med 2008/05/01 | Finak Breast | Fifty-three (53) breast tumor stroma samples and six (6) normal breast stroma samples were analyzed on Agilent 44K microarrays. Sample data includes outcome, recurrence, grade, ER status, PR status, ERBB2 status, lymph node status, age, tumor size, and others. This data was from a dye swap study. The data in Oncomine has been processed by inversing the ratios and averaging the two values per sample. |
| [[46](#_ENREF_46)] | Breast Cancer Res 2009/02/02 | Ma Breast | Twenty (20) ductal breast carcinoma in situ, 18 invasive ductal breast carcinoma, and 28 normal breast samples were analyzed from 14 patients, many with paired epithelial and stromal tissues. Sample data includes estrogen, progesterone and ERBB2 status; age, grade, and N stage. Refer to Ma Breast for corresponding samples that were previously analyzed on cDNA microarrays. |

**References**

1. Pomeroy SL, Tamayo P, Gaasenbeek M, Sturla LM, Angelo M, et al. (2002) Prediction of central nervous system embryonal tumour outcome based on gene expression. Nature 415: 436-442.

2. Stegmaier K, Ross KN, Colavito SA, O'Malley S, Stockwell BR, et al. (2004) Gene expression-based high-throughput screening(GE-HTS) and application to leukemia differentiation. Nat Genet 36: 257-263.

3. Skrzypczak M, Goryca K, Rubel T, Paziewska A, Mikula M, et al. (2010) Modeling oncogenic signaling in colon tumors by multidirectional analyses of microarray data directed for maximization of analytical reliability. PLoS One 5.

4. Sabates-Bellver J, Van der Flier LG, de Palo M, Cattaneo E, Maake C, et al. (2007) Transcriptome profile of human colorectal adenomas. Mol Cancer Res 5: 1263-1275.

5. Cho JY, Lim JY, Cheong JH, Park YY, Yoon SL, et al. (2011) Gene expression signature-based prognostic risk score in gastric cancer. Clin Cancer Res 17: 1850-1857.

6. Logsdon CD, Simeone DM, Binkley C, Arumugam T, Greenson JK, et al. (2003) Molecular profiling of pancreatic adenocarcinoma and chronic pancreatitis identifies multiple genes differentially regulated in pancreatic cancer. Cancer Res 63: 2649-2657.

7. Detwiller KY, Fernando NT, Segal NH, Ryeom SW, D'Amore PA, et al. (2005) Analysis of hypoxia-related gene expression in sarcomas and effect of hypoxia on RNA interference of vascular endothelial cell growth factor A. Cancer Res 65: 5881-5889.

8. Coustan-Smith E, Song G, Clark C, Key L, Liu P, et al. (2011) New markers for minimal residual disease detection in acute lymphoblastic leukemia. Blood 117: 6267-6276.

9. Lee J, Kotliarova S, Kotliarov Y, Li A, Su Q, et al. (2006) Tumor stem cells derived from glioblastomas cultured in bFGF and EGF more closely mirror the phenotype and genotype of primary tumors than do serum-cultured cell lines. Cancer Cell 9: 391-403.

10. Bredel M, Bredel C, Juric D, Harsh GR, Vogel H, et al. (2005) Functional network analysis reveals extended gliomagenesis pathway maps and three novel MYC-interacting genes in human gliomas. Cancer Res 65: 8679-8689.

11. Curtis C, Shah SP, Chin SF, Turashvili G, Rueda OM, et al. (2012) The genomic and transcriptomic architecture of 2,000 breast tumours reveals novel subgroups. Nature 486: 346-352.

12. Turashvili G, Bouchal J, Baumforth K, Wei W, Dziechciarkova M, et al. (2007) Novel markers for differentiation of lobular and ductal invasive breast carcinomas by laser microdissection and microarray analysis. BMC Cancer 7: 55.

13. Quade BJ, Wang TY, Sornberger K, Dal Cin P, Mutter GL, et al. (2004) Molecular pathogenesis of uterine smooth muscle tumors from transcriptional profiling. Genes Chromosomes Cancer 40: 97-108.

14. Nindl I, Dang C, Forschner T, Kuban RJ, Meyer T, et al. (2006) Identification of differentially expressed genes in cutaneous squamous cell carcinoma by microarray expression profiling. Mol Cancer 5: 30.

15. LaTulippe E, Satagopan J, Smith A, Scher H, Scardino P, et al. (2002) Comprehensive gene expression analysis of prostate cancer reveals distinct transcriptional programs associated with metastatic disease. Cancer Res 62: 4499-4506.

16. Lapointe J, Li C, Higgins JP, van de Rijn M, Bair E, et al. (2004) Gene expression profiling identifies clinically relevant subtypes of prostate cancer. Proc Natl Acad Sci U S A 101: 811-816.

17. Grasso CS, Wu YM, Robinson DR, Cao X, Dhanasekaran SM, et al. (2012) The mutational landscape of lethal castration-resistant prostate cancer. Nature 487: 239-243.

18. Taylor BS, Schultz N, Hieronymus H, Gopalan A, Xiao Y, et al. (2010) Integrative genomic profiling of human prostate cancer. Cancer Cell 18: 11-22.

19. Tomlins SA, Mehra R, Rhodes DR, Cao X, Wang L, et al. (2007) Integrative molecular concept modeling of prostate cancer progression. Nat Genet 39: 41-51.

20. Vanaja DK, Cheville JC, Iturria SJ, Young CY (2003) Transcriptional silencing of zinc finger protein 185 identified by expression profiling is associated with prostate cancer progression. Cancer Res 63: 3877-3882.

21. Dyrskjot L, Kruhoffer M, Thykjaer T, Marcussen N, Jensen JL, et al. (2004) Gene expression in the urinary bladder: a common carcinoma in situ gene expression signature exists disregarding histopathological classification. Cancer Res 64: 4040-4048.

22. Liu P, Ramachandran S, Ali Seyed M, Scharer CD, Laycock N, et al. (2006) Sex-determining region Y box 4 is a transforming oncogene in human prostate cancer cells. Cancer Res 66: 4011-4019.

23. Arredouani MS, Lu B, Bhasin M, Eljanne M, Yue W, et al. (2009) Identification of the transcription factor single-minded homologue 2 as a potential biomarker and immunotherapy target in prostate cancer. Clin Cancer Res 15: 5794-5802.

24. D'Errico M, de Rinaldis E, Blasi MF, Viti V, Falchetti M, et al. (2009) Genome-wide expression profile of sporadic gastric cancers with microsatellite instability. Eur J Cancer 45: 461-469.

25. Bhattacharjee A, Richards WG, Staunton J, Li C, Monti S, et al. (2001) Classification of human lung carcinomas by mRNA expression profiling reveals distinct adenocarcinoma subclasses. Proc Natl Acad Sci U S A 98: 13790-13795.

26. Wallace TA, Prueitt RL, Yi M, Howe TM, Gillespie JW, et al. (2008) Tumor immunobiological differences in prostate cancer between African-American and European-American men. Cancer Res 68: 927-936.

27. Crabtree JS, Jelinsky SA, Harris HA, Choe SE, Cotreau MM, et al. (2009) Comparison of human and rat uterine leiomyomata: identification of a dysregulated mammalian target of rapamycin pathway. Cancer Res 69: 6171-6178.

28. Hao Y, Triadafilopoulos G, Sahbaie P, Young HS, Omary MB, et al. (2006) Gene expression profiling reveals stromal genes expressed in common between Barrett's esophagus and adenocarcinoma. Gastroenterology 131: 925-933.

29. Cui JA, Chen YB, Chou WC, Sun LK, Chen L, et al. (2011) An integrated transcriptomic and computational analysis for biomarker identification in gastric cancer. Nucleic Acids Research 39: 1197-1207.

30. Luo JH, Yu YP, Cieply K, Lin F, Deflavia P, et al. (2002) Gene expression analysis of prostate cancers. Molecular Carcinogenesis 33: 25-35.

31. Radvanyi L, Singh-Sandhu D, Gallichan S, Lovitt C, Pedyczak A, et al. (2005) The gene associated with trichorhinophalangeal syndrome in humans is overexpressed in breast cancer. Proceedings of the National Academy of Sciences of the United States of America 102: 11005-11010.

32. Magee JA, Araki T, Patil S, Ehrig T, True L, et al. (2001) Expression profiling reveals hepsin overexpression in prostate cancer. Cancer Research 61: 5692-5696.

33. Roessler S, Jia HL, Budhu A, Forgues M, Ye QH, et al. (2010) A Unique Metastasis Gene Signature Enables Prediction of Tumor Relapse in Early-Stage Hepatocellular Carcinoma Patients. Cancer Research 70: 10202-10212.

34. Skotheim RI, Lind GE, Monni O, Nesland JM, Abeler VM, et al. (2005) Differentiation of human embryonal carcinomas in vitro and in vivo reveals expression profiles relevant to normal development. Cancer Research 65: 5588-5598.

35. Haferlach T, Kohlmann A, Wieczorek L, Basso G, Kronnie GT, et al. (2010) Clinical Utility of Microarray-Based Gene Expression Profiling in the Diagnosis and Subclassification of Leukemia: Report From the International Microarray Innovations in Leukemia Study Group. Journal of Clinical Oncology 28: 2529-2537.

36. Bonome T, Levine DA, Shih J, Randonovich M, Pise-Masison CA, et al. (2008) A gene signature predicting for survival in suboptimally debulked patients with ovarian cancer. Cancer Research 68: 5478-5486.

37. Barretina J, Taylor BS, Banerji S, Ramos AH, Lagos-Quintana M, et al. (2010) Subtype-specific genomic alterations define new targets for soft-tissue sarcoma therapy. Nature Genetics 42: 715-U103.

38. Zhan FH, Barlogie B, Arzoumanian V, Huang YS, Hollmig K, et al. (2006) A gene expression signature of benign monoclonal gammopathy evident in multiple myeloma is linked to good prognosis. Blood 108: 969a-969a.

39. Riker AI, Enkemann SA, Fodstad O, Liu SH, Ren SP, et al. (2008) The gene expression profiles of primary and metastatic melanoma yields a transition point of tumor progression and metastasis. Bmc Medical Genomics 1.

40. Storz MN, van de Rijn M, Kim YH, Mraz-Gernhard S, Hoppe RT, et al. (2003) Gene expression profiles of cutaneous B cell lymphoma. Journal of Investigative Dermatology 120: 865-870.

41. Zhai Y, Kuick R, Nan B, Ota I, Weiss SJ, et al. (2007) Gene expression analysis of Preinvasive and invasive cervical squamous cell carcinomas identifies HOXC10 as a key mediator of invasion. Cancer Research 67: 10163-10172.

42. Su LJ, Chang CW, Wu YC, Chen KC, Lin CJ, et al. (2007) Selection of DDX5 as a novel internal control for Q-RT-PCR from microarray data using a block bootstrap re-sampling scheme. Bmc Genomics 8.

43. Varambally S, Yu JJ, Laxman B, Rhodes DR, Mehra R, et al. (2005) Integrative genomic and proteomic analysis of prostate cancer reveals signatures of metastatic progression. Cancer Cell 8: 393-406.

44. Cutcliffe C, Kersey D, Huang CC, Zeng Y, Walterhouse D, et al. (2005) Clear cell sarcoma of the kidney: Up-regulation of neural markers with activation of the sonic hedgehog and Akt pathways. Clinical Cancer Research 11: 7986-7994.

45. Finak G, Bertos N, Pepin F, Sadekova S, Souleimanova M, et al. (2008) Stromal gene expression predicts clinical outcome in breast cancer. Nature Medicine 14: 518-527.

46. Ma XJ, Dahiya S, Richardson E, Erlander M, Sgroi DC (2009) Gene expression profiling of the tumor microenvironment during breast cancer progression. Breast Cancer Research 11.
